# Atomic Layer Deposition of Metal Oxides and Chalcogenides for High Performance Transistors

**DOI:** 10.1002/advs.202104599

**Published:** 2022-06-16

**Authors:** Chengxu Shen, Zhigang Yin, Fionn Collins, Nicola Pinna

**Affiliations:** ^1^ Institut für Chemie and IRIS Adlershof Humboldt‐Universität zu Berlin Brook‐Taylor‐Str. 2 Berlin 12489 Germany; ^2^ State Key Laboratory of Structural Chemistry Fujian Institute of Research on the Structure of Matter Chinese Academy of Sciences 155 Yangqiao West Road Fuzhou Fujian 350002 China; ^3^ Fujian Science & Technology Innovation Laboratory for Optoelectronic Information of China Fuzhou Fujian 350108 China

**Keywords:** atomic layer deposition, electronics, metal chalcogenides, metal oxides, transistors

## Abstract

Atomic layer deposition (ALD) is a deposition technique well‐suited to produce high‐quality thin film materials at the nanoscale for applications in transistors. This review comprehensively describes the latest developments in ALD of metal oxides (MOs) and chalcogenides with tunable bandgaps, compositions, and nanostructures for the fabrication of high‐performance field‐effect transistors. By ALD various n‐type and p‐type MOs, including binary and multinary semiconductors, can be deposited and applied as channel materials, transparent electrodes, or electrode interlayers for improving charge‐transport and switching properties of transistors. On the other hand, MO insulators by ALD are applied as dielectrics or protecting/encapsulating layers for enhancing device performance and stability. Metal chalcogenide semiconductors and their heterostructures made by ALD have shown great promise as novel building blocks to fabricate single channel or heterojunction materials in transistors. By correlating the device performance to the structural and chemical properties of the ALD materials, clear structure–property relations can be proposed, which can help to design better‐performing transistors. Finally, a brief concluding remark on these ALD materials and devices is presented, with insights into upcoming opportunities and challenges for future electronics and integrated applications.

## Graphical abstract

Atomic layer deposition (ALD) has enabled the development of high‐quality thin film materials in transistors. This review covers recent advances in ALD of metal oxides and chalcogenides with tunable bandgaps, compositions and nanostructures for high‐performance transistor applications. These ALD‐materials show promise as semiconductor channels, transparent electrodes/interlayers, dielectrics, and protecting/encapsulating layers for improving charge transport, switching properties and device stability.

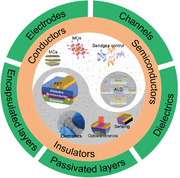

## 1 Introduction

Since the first working transistor was demonstrated in the 1940s, transistors have become a fundamental component of modern electronics. Networks of semiconductor transistors form logic gates essential for the function of microprocessors. Apart from this prominent application, transistors are also widely used in electronic/optoelectronic devices and integrated systems such as flat panel displays, electronic skins, artificial synapses, photodetectors, physical or chemical sensors, and biomedical equipment.^[^ ^1^, ^2^, ^3^, ^4^, ^5^, ^6^, ^7^, ^8^, ^9^, ^10^, ^11^ ^]^ Thus, the application of transistors is ubiquitous within modern digital products.

**Figure 1:**
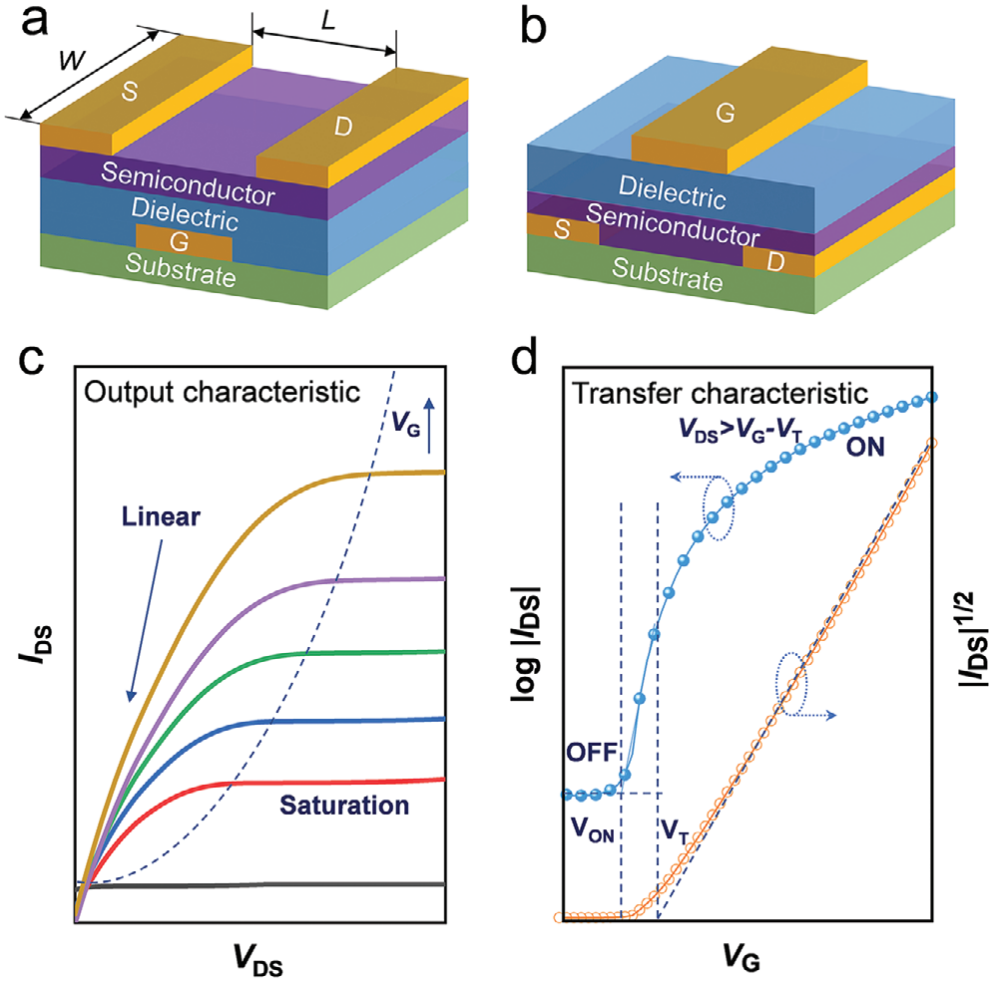
Two common structures of transistors: a) Bottom‐gate device, and b) Top‐gate device. Typical characteristics of transistors: c) Output curves exhibiting linear and saturation operating characteristics, and d) Transfer curve showing the source‐drain current as a function of gate bias.

The basic design of a transistor is composed of three terminals that consist of source (S), drain (D), and gate (G) electrodes. A semiconductor material, also referred to as a channel, contacts the S and D electrodes. A dielectric serves as an insulation layer between the G electrode and the semiconductor layer to electrically isolate the channel. Variation in channel and electrode placement design affords both bottom‐gate and top‐gate structures, which are two commonly used types of transistors (**Figure** **1**a,b). The channel length (*L*) of a transistor is defined as the distance between the S and D regions, and the channel width (*W*) is the total distance across the channel area parallel to the S and D electrodes. Current manufacturing techniques extensively utilize a silicon substrate for transistors in device fabrication of electronic/optoelectronic systems such as processors, communication chips, and image sensors.^[^ ^12^ ^]^ However, flexible substrates such as polyethylene terephthalate (PET), polyimide (PI), polydimethylsiloxane (PDMS), and biodegradable polymers, show good potential for use within the next‐generation flexible devices as a part of wearable/stretchable electronics, and future technologies.^[^ ^9^, ^13^, ^14^, ^15^ ^]^ Most transistors operate using a model based on electro‐magnetic field manipulation. Hence, they are termed field‐effect transistors (FETs).^[^ ^16^, ^17^ ^]^ Application of a voltage between the source and gate of a FET device forms an electric field. This field stimulates the accumulation of charge carriers at the semiconductor/dielectric interface, a process termed capacitive carrier injection. As a result, current flow between S and D electrodes may be modulated according to a gate voltage (*V*_G_, or recorded as *V*_GS_). When *V*_G_ exceeds a threshold voltage (*V*_T_), a conducting channel is established and a source‐drain current (*I*_DS_) can be adjusted by controlling a bias across the S and D electrodes (*V*_DS_). Therefore, transistors can facilitate the adjustment of on‐/off‐state currents by tuning the applied bias voltages, achieving a higher output power than input power. These attributes afford excellent signal conduction, amplification, and switching functions for various applications such as oscillators, photodetectors, and tactile sensors.

The performance of transistors is generally evaluated through charge‐transfer and output characteristics measured for calculating important metrics such as the carrier mobility (*μ*), *V*_T_, on/off current ratio (*I*_ON_/*I*_OFF_), and subthreshold swing (SS). Output curves are obtained by plotting *I*_DS_ as a function of *V*_DS_ whilst a sweeping *V*_G_ is applied (Figure 1c), while transfer curves are illustrated by plotting *I*_DS_ as a function of *V*_G_ at a constant *V*_DS_ (Figure 1d). As the *V*_DS_ applied increases, *I*_DS_ will continue to increase until the channel current saturates. If *V*_DS_ is much lower than the applied *V*_G_ (*V*_DS_ << *V*_G_ − *V*_T_), the transistor operates in a linear regime. At this bias condition, *I*_DS_ increases linearly with *V*_DS_ and charge accumulation across the channel is considered to be evenly distributed. Thus, the current–voltage relationship can be determined by Equation (1),^[^ ^18^ ^]^

(1) $$I_{\mathrm{DS}}=\left(\frac{W}{L}\right)C_{i}\mu_{\mathrm{lin}}\left(V_{G}-V_{T}\right)V_{\mathrm{DS}}$$

where *C*_i_ is the capacitance per unit area of the dielectric, and *μ*_lin_ is the field‐effect mobility in the linear regime. When *V*_DS_ is higher than *V*_G_ − *V*_T_, the conducting channel is pinched off, since the free charge density around the drain contact reduces to nearly (but not quite) zero.^[^ ^19^ ^]^ Under this condition, *I*_DS_ becomes *V*_DS_ independent, and the device operates through a saturation regime. Thus, the carrier mobility of the transistor can be calculated by Equation (2),^[^ ^20^ ^]^

(2) $$I_{\mathrm{DS}}=\left(\frac{W}{2L}\right)C_{i}\mu_{\mathrm{sat}}{\left(V_{G}-V_{T}\right)}^{2}$$

where *μ*_sat_ is the mobility in the saturated regime. Transfer curves reflect the conditions of a saturated regime via a plot of *I*_DS_^1/2^ against *V*_G_ (Figure 1d). This relationship yields a straight line, where the square of its slope is proportional to the charge carrier mobility. Meanwhile, *V*_T_ can be obtained by extrapolation to an intercept of the linear part of the *I*_DS_^1/2^‐*V*_G_ plot. Considering that the determination of *V*_T_ is sometimes ambiguous, one can employ *V*_ON_ as a parameter for describing the *V*_G_ needed to turn the device on, that is, the potential at which *I*_DS_ starts to flow because of field‐induced charge accumulation at the semiconductor/dielectric interface.^[^ ^21^ ^]^ The on‐state and off‐state currents of FETs can be obtained from the transfer characteristics to afford *I*_ON_/*I*_OFF_, which directly reflects the control of an applied gate bias over the conductive channel. The SS is defined as the inverse of the maximum slope of the logarithmic *I*_DS_ plot (expressed as V/decade) and can be extracted from a transfer curve based on the relationship from Equation (3).^[^ ^16^ ^]^

(3) $$\mathrm{SS}={\left(\frac{d\log I_{\mathrm{DS}}}{dV_{G}}\right)}_{\max}^{-1}$$

Note that lower SS values lead to higher switching speeds and lower power consumption. Thus, low values of SS (<< 1) are desirable for improving the ratio between on‐ and off‐currents and making the FET more energy efficient.^[^ ^16^, ^19^ ^]^

Beyond these key parameters, the operating voltages and device stability are also important for practical applications of transistors. FETs based on SiO_2_ dielectrics suffer from high‐operating voltages with tens or hundreds of volts. This limits application in wearable devices, where safety and low power consumption are important factors for adoption. For example, low voltage (<5 V), flexible FETs have been demonstrated through the incorporation of high‐capacitance polyelectrolytes as gate dielectrics.^[^ ^22^, ^23^, ^24^ ^]^ Semiconductor materials and processing methods largely determine the performance of FETs. Elemental semiconductors such as amorphous silicon (*a*‐Si) and polycrystalline silicon (*poly*‐Si) were commonly employed for FETs in the electronic industry.^[^ ^1^ ^]^ The development of novel semiconductor materials using inorganic compounds, organic conjugated molecules, quantum dots, and 2D nanomaterials for high‐performance FETs such as metal–oxide–semiconductor FETs (MOSFETs), organic FETs (OFETs), quantum dot FETs (QFETs), show promising results for integration within next‐generation devices.^[^ ^19^, ^25^, ^26^, ^27^ ^]^ Inorganic group‐VI (O, S, Se, and Te) materials are recognized as excellent semiconductor candidates for FETs within dedicated applications such as transparent electronics, large‐area thin film electronics, active matrix displays, inverters, ring oscillators, and integrated circuits.^[^ ^16^, ^21^, ^28^ ^]^ Several examples of metal oxides (MOs) and metal chalcogenides (MCs) have attracted remarkable attention in the development of thin‐film transistors (TFTs) and emerging applications,^[^ ^10^, ^29^, ^30^, ^31^ ^]^ indicating good potential for the development of high performance electronic/optoelectronic devices as well as integrated systems with state‐of‐the‐art silicon electronic and photonic devices.

Beyond innovation of semiconductor materials, research and development of transistors have also been driven by the fast‐developing application of fabrication technologies such as solution‐processing, thermal evaporation, sputtering, chemical vapor deposition (CVD), atomic layer deposition (ALD), and many others.^[^ ^42^, ^48^, ^49^, ^50^, ^51^ ^]^ **Table** **1** briefly compares the advantages and disadvantages of these different film growth techniques. In particular, ALD is recognized for producing thin‐films with good conformality and reproducibility. These attractive features are beneficial for the fabrication of high‐quality semiconductor films and dielectric layers in FETs at the nanoscale.^[^ ^52^ ^]^

**Table 1:** Comparison of several typical film growth techniques utilized for transistor fabrication

Technique	ALD	CVD	Solution processing	Sputtering	Thermal evaporation
Step coverage	Excellent	Good	Poor	Moderate	Moderate
Fine thickness control	Excellent	Good	Moderate	Moderate	Good
Film uniformity	Excellent	Good	Moderate	Poor	Good
Interface quality	Excellent	Good	Moderate	Moderate	Good
Processing temperature	Low	High	Low	Low	Moderate
Compatibility with flexible substrate	Good	Poor	Excellent	Good	Moderate
Large‐area scalability	Moderate	Moderate	Excellent	Moderate	Good
Growth rate	Low	Moderate	High	High	Moderate
Manufacturing cost	High	Moderate	Low	Moderate	Moderate

**Figure 2:**
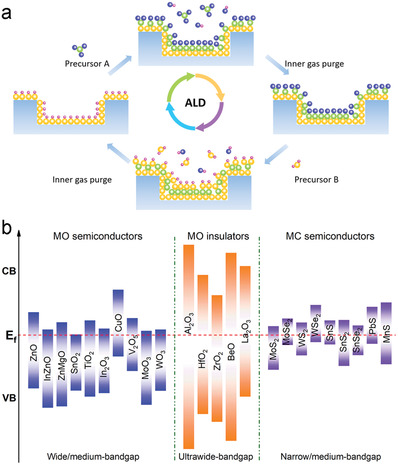
a) Schematic illustration of a typical ALD cycle for growing inorganic films. b) Schematic positions of the valence band (VB) and conduction band (CB) relative to the Fermi level (E_f_) for MO semiconductors and insulators, and MC semiconductors made by ALD techniques in previous reports.^[^ ^32^, ^33^, ^34^, ^35^, ^36^, ^37^, ^38^, ^39^, ^40^, ^41^, ^42^, ^43^, ^44^, ^45^, ^46^, ^47^ ^]^ The red dash line indicates the E_f_.

An ALD protocol is comprised of a series of self‐limiting surface reactions between gaseous precursors at the interface of a solid substrate.^[^ ^53^, ^54^ ^]^ Alternating pulse and purge sequences of different precursor reactants supplied using an inert carrier gas ensure that only selective self‐limiting reactions occur (**Figure** **2**a). The sequential nature isolates reactants, which greatly enhances control over the chemisorption reaction at surface‐active growth sites. In this manner, the film thickness may be tuned according to the number of ALD cycles, affording angstrom level precision.^[^ ^55^ ^]^ In addition, low growth temperatures and the conformal coating capabilities of ALD make it compatible with a greater variety of flat and curved substrates.^[^ ^56^, ^57^, ^58^, ^59^ ^]^ The demonstration of ALD in fabricating numerous types of thin‐film materials including pure elements, oxides, chalcogenides, carbides, nitrides, and phosphates on various rigid or flexible supports, highlights its adaptability.^[^ ^60^, ^61^, ^62^, ^63^ ^]^ A variety of bandgap‐tunable MO materials have been fabricated using ALD techniques for applications as semiconductor channels, electrodes, and electrode interlayers in TFTs.^[^ ^64^, ^65^, ^66^, ^67^ ^]^ Further adaptations of ALD have yielded MO insulators integrated as gate dielectrics and encapsulating layers in FETs.^[^ ^68^, ^69^, ^70^ ^]^ An increased focus on 2D materials for applications within FETs has stimulated further interest in layered materials such as transition metal chalcogenides (TMCs).^[^ ^28^, ^31^, ^71^, ^72^ ^]^ Furthermore, ALD‐fabricated TMC semiconductors have been combined with conventional silicon‐based devices to produce hybrid devices, enabling the integration of flexible designs.^[^ ^73^, ^74^ ^]^ The large variety of semiconductor materials (Figure 2b) fabricated by ALD, indicates its potential as a transistor component manufacturing method. Thus, the integration of ALD‐MOs and MCs within FETs may serve to improve the performance of existing designs, as well as provide complementary and novel properties for next‐generation electronic/optoelectronic devices.

In this article, we aim to present recent advances in the transistor‐based applications of ALD MOs and MCs. Emphasis is placed on the tunability of materials using ALD, the influence of ALD on the performance optimization of FETs as well as device applications. The review will first explore the application of ALD for various MO materials used as channel, dielectric and encapsulating materials within FETs, as well as electrode and electrode interlayers. The latter section will discuss MC materials as high‐performance semiconductors, with a focus on their electrical performance within FETs and possible heterostructure devices. Last, we will conclude with a brief remark on the merits and limits of ALD‐MOs/MCs for transistors as well as their opportunities and challenges in future electronics.

## 2 ALD of Metal Oxides for FETs

Over the past decades, MOs have gained attention for electronic applications due to their tunable structures, unique properties, and facile processing methods.^[^ ^14^, ^75^ ^]^ MOs are generally grouped as semiconductors or insulators, according to the differences in band position and bandgaps.^[^ ^66^, ^76^, ^77^ ^]^ Recently, uniform MO films with precisely controlled thicknesses and adjustable properties were deposited by ALD,^[^ ^78^ ^]^ which performed promisingly upon integration within FET devices.^[^ ^79^ ^]^

### 2.1 Metal Oxide Semiconductors

MO semiconductors, with tunable bandgap values from 1 to 3 eV, are promising transparent channel materials that have attracted great interest in the microelectronics industry.^[^ ^64^, ^65^, ^80^ ^]^ Binary MO semiconductors deposited by ALD, such as ZnO,^[^ ^64^, ^81^ ^]^ In_2_O_3_,^[^ ^65^ ^]^ SnO,^[^ ^80^ ^]^ and TiO_2_,^[^ ^82^ ^]^ have exhibited transport and switching properties meeting the prerequisites for applications in FETs. Besides, multinary MO semiconductors such as indium zinc oxide (IZO),^[^ ^83^ ^]^ and indium gallium zinc oxide (IGZO),^[^ ^84^ ^]^ can be effectively fabricated by facile modification of existing ALD processes. Compared with binary MOs used for FETs, multinary MO semiconductors have been demonstrated to deliver more attractive device performance, such as higher mobility, better stability, lower leakage current, and smaller SS.^[^ ^83^, ^84^, ^85^ ^]^ In addition to the success of n‐type MO semiconductors, p‐type MO materials such as SnO, CuO, and Cu_2_O as well as emerging alternatives, are also involved in this review due to their significant roles in new‐generation electronics. **Table** **2** summarizes key electrical performance parameters of some representative FETs based on various MO semiconductors fabricated using ALD under different conditions.

**Table 2:** Device characteristics of representative FETs using semiconducting MOs synthesized by ALD techniques

Material	Method	*T*_ALD_ [°C]	Precursors^a)^	*μ* [cm^2^ V^−1^s^−1^]		*I*_ON_/*I*_OFF_	SS [V dec^−1^]	*V*_T_ [V]	Ref.
				n‐type	p‐type				
ZnO	ALD	170	DEZn + H_2_O	17	–	10^8^	–	–	[86]
	ALD	100	DEZn + NH_3_•H_2_0	–	Hall	10^5^	–	–	
	ALD	≤110	DEZn + H_2_O	20.2	–	10^5^	0.38	2.4	[87]
	SALD	200	DEZn + H_2_O with NH_3_	≈17	–	–	–	–	[81]
	PEALD	200	–	≈11.3	–	–	–	2.2	[88]
InO_x_	ALD	150	InCA‐1 + H_2_O_2_	≈10	–	10^9^	0.63	−0.2	[79]
	PEALD	100–250	Et_2_InN(SiMe_3_)_2_ + O_2_ plasma	39.2	–	–	0.27	–	[89]
In_2_O_3_	ALD	225	TMIn + H_2_O	≈4	–	10^10^	0.13	–	[90]
	ALD	300	DADI + ozone	41.8	–	10^7^	0.1	−0.8	[65]
SnO_2_	ALD	–	TMT + H_2_O plasma	8.23 × 10^−5^	–	–	–	−3.5	[91]
	PEALD	60	Sn(DMP)_4_ + O_2_ plasma	12	–	10^7^	–	–	[75]
	PEALD	70–130	Sn(dmamp)_2_ + O_2_ plasma	6.24	–	10^6^	–	1.88	[92]
SnO	ALD	150–210	Sn(dmamp)_2_ + H_2_O	–	≈1	2 × 10^6^	1.8	–	[93]
	ALD	200	Sn(dmamp)_2_ + H_2_O	–	1.6	1.2 × 10^5^	1.06	–	[80]
CuO	ALD	100	(hfac)Cu‐(I)(DMB) + O_3_	–	5.64	1.8 × 10^5^	0.75	1.88	[94]
Cu_2_O	PEALD	160–240	Cu(^5^Bu‐Me‐amd)]_2_ + O_2_ plasma	–	0.1	2 × 10^3^	–	–	[95]
TiO_2_	ALD	150	TDMAT + H_2_O	0.67	–	2.5 × 10^6^	0.35	6.5	[96]
	ALD	250	TDMAT + H_2_O	–	400	–	–	–	[82]
InSnO	ALD	225	TMIn/TDMASn + H_2_O	28	–	3.3 × 10^7^	0.08	0.33	[97]
InZnO	SALD	160	DEZn/TMIn + O_2_ plasma	32	–	–	0.25	–	[83]
NbZnO	ALD	175	DEZn/Nb(OEt)_5_ + H_2_O	7.9	–	10^8^	0.34	8.5	[98]
InGaZnO	SALD	200	DEZn/TMIn/TEGa + H_2_O	≈3.5	–	≈10^3^	–	≈−22.5	[84]
	ALD	200	DEZn/DADI/TMGa + O_2_ plasma	≈74.3	–	8.9 × 10^8^	0.26	−1.3	[99]
MgZnO	ALD	200	Mg(CpEt)_2_/DEZn + H_2_O	3.6	–	7 × 10^6^	0.8	8.1	[100]
AlZnO	ALD	150	DEZn/TMA + H_2_O	≈6	–	–	–	2.25	[101]
HfZnO	ALD	200	TEMAH/DEZn + H_2_O	4.2	–	–	0.52	−4.3	[102]

^a)^ Note that DEZn is diethylzinc; InCA‐1 is [1,1,1‐trimethyl‐N‐(trimethylsilyl)silanaminato] indium; DADI is (3‐(dimethylamino)propyl) dimethylindium; Et_2_InN(SiMe_3_)_2_ is diethyl[bis(trimethylsilyl) amido]indium; TMT is tetramethyltin; (hfac)Cu‐(I)(DMB) is hexafluoroacetyl‐acetonate Cu(I) (3,3‐dimethyl‐1‐butene); TDMAT is tetrakis (dimethylamido) titanium(IV); Cu(^5^Bu‐Me‐amd)]_2_ is bis(N,N’‐di‐sec‐butylacetami‐dinato)dicopper(I); TMIn is trimethylindium; TDMASn is tetrakis(dimethylamino)tin; TEGa is triethyl gallium; TMGa is trimethylgallium; Nb(OEt)_5_ is niobium pentaethoxide; Mg(CpEt)_2_ is bis(ethylcyclopentadienyl) magnesium; TMA is trimethylaluminum; TEMAH is tetrakisethylmethylamino‐hafnium.

#### 2.1.1 Zinc Oxide

Due to a wide‐bandgap of 3.37 eV, zinc oxide (ZnO) is a promising transparent oxide semiconductor and attracts great attention for applications in solar cells and TFTs.^[^ ^103^, ^104^ ^]^ ZnO‐based TFTs exhibit notable performance characteristics such as a high *I*_ON_/*I*_OFF_ ratio of 10^8^ and a mobility of 80 cm^2^ V^−1^s^−1^.^[^ ^86^, ^105^, ^106^, ^107^ ^]^ Synthesis of ZnO by ALD commonly uses diethyl zinc (DEZ) as a precursor.^[^ ^87^, ^108^ ^]^ ALD‐fabricated ZnO films deposited at a low reaction temperature (80 to 250 °C), exhibit excellent *I*_ON_/*I*_OFF_ (10^9^) and mobility (50 cm^2^ V^−1^s^−1^) metrics.^[^ ^86^, ^109^ ^]^ However, deposition temperature greatly influences the electrical properties of ALD‐ZnO films.^[^ ^110^ ^]^ Mobility decreases at low deposition temperatures, in contrast, the *I*_ON_/*I*_OFF_ increases.^[^ ^109^ ^]^ High deposition temperatures can generate greater numbers of defects such as oxygen vacancies, resulting in increased carrier mobility of ALD‐ZnO.^[^ ^111^ ^]^ Correspondingly, oxide film defects resulting from low temperatures can be passivated by O‐H species, which will reduce the mobility, but increase the *I*_ON_/*I*_OFF_.^[^ ^112^ ^]^ Generally, ALD‐ZnO films fabricated using higher deposition temperatures exhibit higher carrier concentrations (≥10^19^), much higher than the appropriate carrier concentration of ≈10^14^–10^17^ for the traditional MO channel layer.^[^ ^111^ ^]^

It was reported that post‐annealing in an oxygen atmosphere could efficiently reduce the carrier concentration, thus obtaining high‐quality ZnO films with appropriate semiconductor‐related properties.^[^ ^113^, ^114^ ^]^ For instance, the electrical performance of ALD‐ZnO films deposited at a relatively high temperature (≈200–250 °C), is improved by a post‐annealing at 300 °C in O_2_.^[^ ^110^ ^]^ The annealed ZnO films exhibit similar transfer characteristics as films fabricated at lower temperatures. Besides the high‐temperature‐deposited films, Bang et al. also improved the electrical performance of low‐temperature‐deposited ZnO films via higher temperature post‐annealing treatment.^[^ ^114^ ^]^ The ALD‐ZnO film deposited at 100 °C using DEZ, showed good device performance with a mobility of 1.2 cm^2^ V^−1^ s^−1^ and an *I*_ON_/*I*_OFF_ of 3.1 × 10^6^, which can be further improved to 1.8 cm^2^ V^−1^ s^−1^ and 1.7 × 10^7^, respectively, after post‐annealing the ALD‐ZnO film at 250 °C in ambient air. As a consequence, the SS decreased from 0.53 to 0.34 V dec^−1^. The enhanced electrical performance of ZnO transistors can be attributed to the formation of a Zn‐rich phase between the semiconductor layer and the metal electrode during the post‐annealing treatment, which may increase the carrier concentration at the metal/semiconductor interface and decrease the contact resistance. Higher annealing temperatures cause faster diffusion of metal atoms, resulting in a rougher interface. Thus, the highly conducting Zn‐rich phase may alter the length of the semiconducting channel.

**Figure 3:**
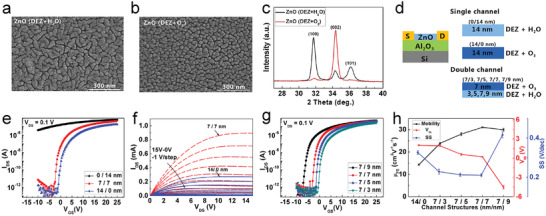
a,b) Scanning electron microscope (SEM) images and c) XRD patterns of ALD‐ZnO films derived from H_2_O (a) and O_3_ (b) as the reactant agent, respectively. d) Schematic of ALD‐ZnO TFTs with the single and double channel structures. e) Transfer and f) output curves of ALD‐ZnO TFTs with the single and bilayer channel structures. g) Transfer characteristics of the bilayer ALD‐ZnO TFTs with varying thicknesses of the H_2_O‐derived ZnO interlayer. h) Trend in mobility (black), *V*_T_ (red), and SS (blue) of the ALD‐ZnO TFTs with different channel structures. Reproduced with permission.^[^ ^115^ ^]^ Copyright 2020, Elsevier.

In addition to the deposition temperature, oxygen source is another critical factor that affects the quality of ALD‐ZnO films. Strong oxidants such as O_3_ yield smaller average grain sizes in comparison to H_2_O in application with DEZ (**Figure** **3**a,b).^[^ ^115^ ^]^ X‐ray diffraction (XRD) analysis shows additional changes in the preferred grain orientation of ZnO film, indicating the tunability of growth direction according to oxidant selection (Figure 3c). The two ALD‐ZnO films and their bilayer structure can be employed as n‐type channels in TFTs (Figure 3d). Analysis of their individual transfer curves clearly indicates that the O_3_‐derived ZnO film shows better electrical performance than the H_2_O‐derived ZnO film (Figure 3e). Moreover, by using the H_2_O‐derived ZnO interlayer to decrease the interfacial trap density, the resulting bilayer ALD‐ZnO channel shows improved electrical performance relative to the single layer channel (Figure 3e–h). Upon increase to the interlayer thickness, the SS of the TFTs decreases while the mobility increases significantly. However, when its thickness exceeds 7 nm, the electrical performance starts to degrade again (Figure 3g–h). As result, the optimized bilayer ALD‐ZnO TFTs deliver a highly improved mobility of 31.1 cm^2^ V^−1^ s^−1^ with a low *V*_T_ of 0.14 V, a large *I*_ON_/*I*_OFF_ of 10^8^, a small SS of 0.21 V dec^−1^, and a good positive bias stress stability.

Assisted by the generation of oxygen vacancies, ALD‐ZnO films are considered intrinsic n‐type semiconductors. However, Guziewicz et al. has demonstrated successfully the deposition of p‐type ZnO semiconductors through in situ nitrogen doping.^[^ ^86^ ^]^ During the ALD process, an ammonia water solution was used as an oxygen source instead of pure water or oxygen, and the film was annealed briefly in a nitrogen atmosphere after deposition. As a result, the N‐doped ZnO exhibited a p‐type characteristic with a Hall carrier concentration of ≈10^18^ cm^−3^. Moreover, a p‐n homojunction was demonstrated by depositing an n‐type ZnO layer onto the p‐type ZnO, showing an *I*_ON_/*I*_OFF_ close to 10^5^.

ALD‐ZnO also shows good potential for application within transparent and flexible electronics because of its high transmittance and low‐temperature processing requirements.^[^ ^87^, ^103^ ^]^ For instance, ZnO‐based transparent TFTs with good optical transmission in the visible range were successfully fabricated using ALD on transparent glass substrates at a temperature below 100 °C.^[^ ^103^ ^]^ Separated by a dielectric of ALD‐Al_2_O_3_/HfO_2_/Al_2_O_3_, the ALD‐ZnO film acted as both a channel material and a gate electrode. Flexible TFTs using the ALD‐ZnO channel and TiO_2_/Al_2_O_3_ passivated layers were also achieved on plastics at low temperatures.^[^ ^87^, ^88^ ^]^ The flexible ALD‐ZnO TFTs showed outstanding electrical performance with electron mobility of ≈17 cm^2^ V^−1^ s^−1^, an *I*_ON_/*I*_OFF_ of 10^5^, and a SS of 0.4 V dec^−1^.^[^ ^87^ ^]^

**Figure 4:**
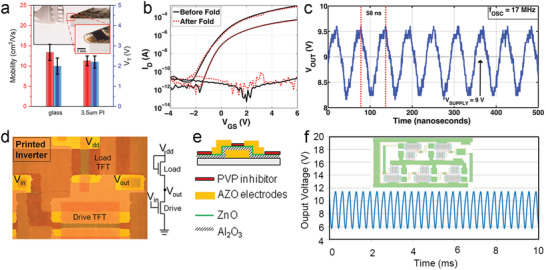
a) Performance comparison of PEALD‐ZnO TFTs based on glass and PI substrates, with data collected from the Ref. [88]. The inset is a photograph of the ZnO TFTs on a 3.5 µm PI substrate folded with a bending radius of ≤ 500 µm. b) Transfer curves of the corresponding device before (black) and after (red) folding. c) Output waveform of the flexible TFT oscillator based on ALD‐ZnO on the PI substrate. Reproduced with permission.^[^ ^88^ ^]^ Copyright 2016, Wiley VCH. d) Optical image of printed inverter with a schematic logic circuit. e) Device structure of the printed TFTs. f) Output waveform of the fabricated five‐stage ring oscillator with an input voltage of 20 V (the inset shows a layout of the fabricated devices). Reproduced with permission.^[^ ^81^ ^]^ Copyright 2015, American Chemistry Society.

With the use of plasma‐enhanced ALD (PEALD), flexible ZnO‐based TFTs have been fabricated via the deposition of ZnO thin‐films onto PI substrates.^[^ ^88^ ^]^ The TFTs deposited on a 3.5 µm flexible PI substrate exhibit comparable electron mobility (1.3 ± 1.2 cm^2^ V^−1^ s^−1^) and *V*_T_ (2.2 ± 0.3 V) values to those of TFTs on a glass substrate (**Figure** **4**a). Investigation of device performance before and after fold‐induced mechanical stress (Figure 4b), shows a negligible degradation of mobility and *V*_T_. The practical application of ZnO‐based TFTs was further demonstrated using a cross‐coupled LC oscillator circuit, with the flexible PI substrate outperforming analogous TFTs deposited onto glass. Using a *V*_supply_ of 9 V, the oscillation frequency (*f*_OSC_) measured 17 MHz (Figure 4c), well above the cutoff frequency (*f*_T_) for operation on glass substrates (12.9 MHz).

Printed inverters using ZnO‐based TFTs (Figure 4d) were prepared using a modified version of ALD, which applies spatial isolation techniques to achieve ALD (SALD).^[^ ^81^ ^]^ The fabricated TFTs (Figure 4e) exhibited excellent electrical performance with high mobility (15 cm^2^ V^−1^ s^−1^). Furthermore, inverters arranged within a five‐stage, enhancement‐mode ring oscillator architecture displayed a frequency response of 2.68 kHz at an input voltage of 20 V (Figure 4f). In conclusion, the tunable nature of ALD has afforded ZnO semiconductors with strong electrical performance upon integration within TFTs. Its compatibility with flexible substrates, along with its performance within integrated circuits, reflects its promise for use within more complex device architectures.

#### 2.1.2 Indium Oxide

Investigation into heavier MOs has demonstrated that indium oxide (In_2_O_3_) possesses excellent properties as a wide bandgap (≈3.6 eV) semiconducting/conducting material.^[^ ^65^, ^116^ ^]^ Deposition of In_2_O_3_ using low‐temperature ALD yields highly optically transparent (>85%) films, which perform moderately well according to mobility (15 cm^2^ V^−1^ s^−1^) and *V*_T_ (≈−0.2 V) metrics.^[^ ^116^ ^]^

Films grown using PEALD with the precursor diethyl[bis(trimethylsilyl)amido]indium [Et_2_InN(SiMe_3_)_2_] and O_2_ plasma (100–250 °C) resulted in polycrystalline In_2_O_3_, which upon the application within a TFT displayed a significantly better mobility (39.2 cm^2^ V^−1^ s^−1^).^[^ ^89^ ^]^ The low voltage threshold and high mobility suggest ALD‐In_2_O_3_ is an extremely promising candidate for transparent TFT‐containing devices.^[^ ^117^ ^]^ However, In_2_O_3_ films grown at low temperatures typically form non‐stoichiometric amorphous films with carrier concentrations analogous to metal‐like materials, likely resulting from excess oxygen vacancy site formation.^[^ ^79^ ^]^ Simulations applying density functional theory (DFT) suggest increasing the order within In_2_O_3_ thin films and consequently, the stoichiometric nature of the film leads to an attenuation in the metal‐like conductivity.^[^ ^118^ ^]^ These findings are also supported experimentally. Transmission electron microscopy (TEM) analysis indicates that as‐deposited amorphous In_2_O_3_ undergoes transformation upon post‐deposition annealing at 300 °C within an oxygen atmosphere, resulting in crystalline In_2_O_3_.^[^ ^65^ ^]^ The annealed In_2_O_3_ films exhibited improved electrical performance within TFT devices. The mobility was increased from 20.12 to 41.8 cm^2^ V^−1^ s^−1^, the SS was decreased from 400 to 100 mV dec^−1^, and the *V*_T_ was reduced from −6.7 V to −0.8 V.

**Figure 5:**
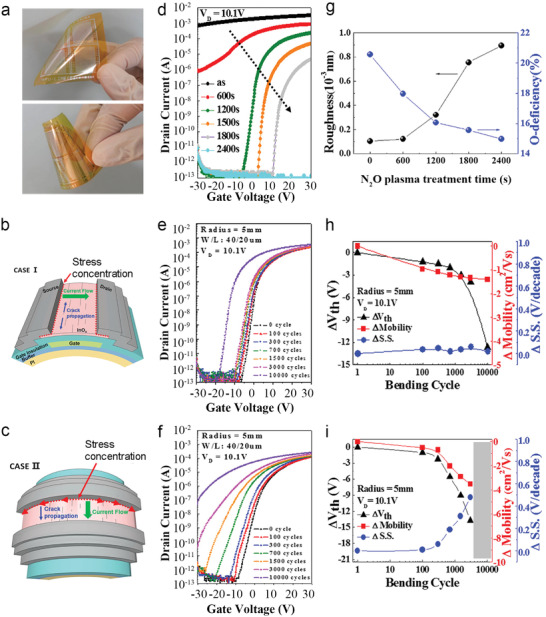
a) Photograph of the flexible ALD‐InO_x_ TFTs based on PI substrates. b,c) Schematic of InO_x_ TFTs with two bending axes along the channel width (b, case I) and the channel length (c, case II), respectively. d) Transfer curves of TFTs based on the ALD‐InO_x_ with different N_2_O plasma time. e,f) Transfer curves and h,i) the corresponding change in *V*_T_, mobility and SS at different bending cycles of flexible InO_x_ TFTs with case I bending (e and h) and with case II bending (f and i). g) Change in the surface roughness and O‐deficiency of InO_x_ films with an increase of N_2_O plasma treatment time. Reproduced with permission.^[^ ^79^ ^]^ Copyright 2016, American Chemistry Society

In another study, amorphous InO_x_ deposited at 150 °C was post‐treated with N_2_O plasma under different exposure times (600–2400 s) to investigate the effects on device performance (**Figure** **5**).^[^ ^79^ ^]^ Prolonged plasma exposure leads to an increase in roughness of the InO_x_ surface and a considerable decrease in O‐deficiency (Figure 5g). The TFTs made by using the plasma‐treated InO_x_ films transit from a metal‐like conductor to a semiconducting device. The switching performances such as the *I*_ON_/*I*_OFF_ are greatly improved with an increase in the plasma time (Figure 5d). The low‐temperature approach enabled further investigation into flexible ALD‐InO_x_ TFTs through deposition on a PI substrate (Figure 5a). Repetitive bending tests were carried out to evaluate the electrical performance of the flexible TFTs with two different bending axes, along the channel length (case I, Figure 5b) and along the channel width (case II, Figure 5c), respectively. For case I, the *V*_T_ shifts gradually in a negative direction after 3000 bending cycles, whilst the mobility and SS exhibit only slight changes (Figure 5e,h). However, more dramatic changes were measured for case II. After 700 bending cycles, the mobility and *V*_T_ decrease dramatically, while the SS increases drastically (Figure 5f,i). These results suggest that ALD‐fabricated InO_x_ films may be suitable for integration within flexible TFT devices. However, the TFT architecture influences durability significantly. Furthermore, both methods demonstrating stoichiometric enrichment of amorphous InO_x_ utilize oxygen enrichment post‐processing techniques at significantly different temperatures, suggesting the barrier of formation for In_2_O_3_ is kinetic in nature. Further mechanism investigation may assist in supporting this correlation as well as assisting in the optimization of future ALD protocols.

#### 2.1.3 Tin Oxides

Tin dioxide (SnO_2_), a wide bandgap (≈3.6 eV) n‐type semiconductor, exhibits excellent electrical properties such as large electron mobilities (≤147 cm^2^ V^−1^ s^−1^) and high *I*_ON_/*I*_OFF_ ratios (10^7^).^[^ ^75^, ^119^, ^120^, ^121^ ^]^

**Figure 6:**
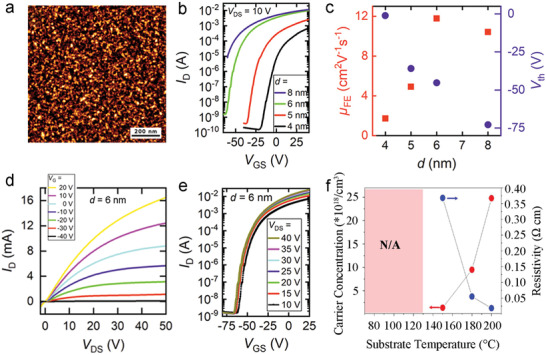
a) Atomic force microscope (AFM) images of an ALD‐SnO_2_ film deposited at 60 °C. b) Transfer characteristics of SnO_2_‐TFTs with different thicknesses of the ALD‐SnO_2_ channel. c) Thickness‐dependent mobility (red) and *V*_T_ (blue) of the ALD‐SnO_2_ TFTs. d) Output and e) transfer curves of the optimized TFT based on ALD‐SnO_2_ (6 nm) at different applied voltages. Reproduced with permission.^[^ ^75^ ^]^ Copyright 2019, American Chemistry Society. f) Change in the carrier concentration (red) and resistivity (blue) of ALD‐SnO_2_ with deposition temperatures. Reproduced with permission.^[^ ^92^ ^]^ Copyright 2012, Elsevier.

Deposition of high‐quality SnO_2_ films may be achieved using a range of tin precursors such as TDMASn,^[^ ^66^, ^122^ ^]^ dimethylamino‐2‐methyl‐2‐propoxy‐tin(II) [Sn(dmamp)_2_],^[^ ^92^ ^]^ and tetrakis‐(dimethylamino)propyl tin(IV) [Sn(DMP)_4_].^[^ ^75^ ^]^ SnO_2_ films deposited using Sn(DMP)_4_ and oxygen plasma at low temperatures (60 °C) feature small hillocks (a low surface roughness value of 0.22 nm, **Figure** **6**a).^[^ ^75^ ^]^ Evaluation of transfer characteristics within bottom‐gate TFTs indicates that the film thickness greatly influences mobility and *V*_T_ (Figure 6b,c). In thicker layers, the bulk SnO_2_ remains in a non‐depleted state, causing parallel conduction. In comparison, thin films suffer from surface roughness scattering. However, at an optimized film thickness (6 nm), the TFTs display typical n‐type output and transfer characteristics (Figure 6d,e), yielding an electron mobility of 12 cm^2^ V^−1^ s^−1^ and an *I*_ON_/*I*_OFF_ of 10^7^. The deposition temperature of ALD‐SnO_2_ has also been demonstrated to influence electrical performance.^[^ ^92^ ^]^ Improvements in channel mobility (2.31 to 6.24 cm^2^ V^−1^ s^−1^) and *V*_T_ (7.47 to 1.88 V) are observed upon increasing deposition temperature (70 to 130 °C). Above 130 °C, carrier concentration increases in tandem with a decrease in resistivity (Figure 6f), resulting in conductor characteristics. By contrast, room‐temperature ALD‐SnO_2_ using tetramethyltin [Sn(CH_3_)_4_] and plasma‐excited humidified argon, can also be used as a channel material to display n‐type behaviors but with pretty low mobility (8.23 × 10^−5^ cm^2^ V^−1^ s^−1^).^[^ ^91^ ^]^

**Figure 7:**
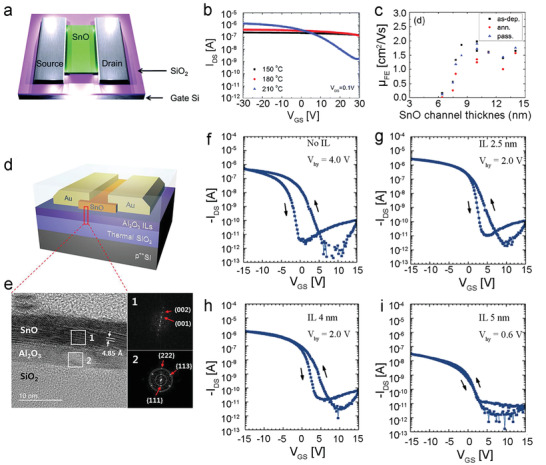
a) Schematic device of an ALD‐SnO TFT. b) Transfer characterization of TFTs based on ALD‐SnO channel films with different ALD reaction temperatures. c) Change in field‐effect mobility of TFTs with different SnO channel thicknesses. Reproduced with permission.^[^ ^93^ ^]^ Copyright 2017, Royal Society of Chemistry. d) Schematic and e) cross‐section high resolution transmission electron microscope (HRTEM) image of the TFT based on ALD‐SnO with an Al_2_O_3_ interlayer. f–i) Transfer curves of ALD‐SnO TFTs without (f), and with the 2.5 (g), 4 (h), and 5 nm (i) interlayers. Reproduced with permission.^[^ ^80^ ^]^ Copyright 2019, Wiley VCH.

Differing from SnO_2_, tin monoxide (SnO) behaves as a p‐type semiconductor with a wide bandgap (≈3.0 eV) and offers a moderate field‐effect mobility (≈6.75 cm^2^ V^−1^ s^−1^).^[^ ^123^, ^124^ ^]^ Although a variety of studies on tin oxides synthesized by ALD have been reported, it is still a challenge to obtain high‐quality p‐type SnO films.^[^ ^80^, ^93^ ^]^ Han et al. demonstrated tunability of Sn/O film composition through the use of different oxygen‐containing co‐reactants.^[^ ^123^ ^]^ Reactions using a strong oxidant such as O_3_ or O_2_ plasma yielded n‐type SnO_2_ films, whilst H_2_O afforded SnO films. Deposition temperature also influenced morphology with higher temperatures (150–210 °C) resulting in greater SnO crystallinity and corresponding improvements to the electrical performance of ALD‐SnO TFTs (**Figure** **7**a,b).^[^ ^93^ ^]^ The increased *I*_ON_/*I*_OFF_ is mainly attributed to effective reduction of extrinsic hole concentrations in the SnO films, while the improved mobility may result from the increased grain size of ALD‐SnO films grown at relatively high temperatures. Optimization of TFT channel thickness using ALD demonstrated further improvements in TFT performance (Figure 7c). The optimized ALD‐SnO TFTs exhibited interesting electrical performance with a hole mobility of ≈1 cm^2^ V^−1^ s^−1^, an *I*_ON_/*I*_OFF_ of 2 × 10^6^, and a SS of 1.8 V dec^−1^. Diffusion of Sn(IV) into the SiO_2_ insulator layer forms trap sites during both ALD and post‐annealing, leading to poor device performance. Adoption of an insulating Al_2_O_3_ interfacial layer (IL) between the SiO_2_ gate dielectric and ALD‐SnO channel layer reduces trap site density (Figure 7d,e).^[^ ^80^ ^]^ Comparison of Al_2_O_3_ IL thickness (Figure 7f–i) highlights the effects of ILs upon hysteresis voltage (*V*_hy_), with significant attenuation of *V*_hy_ observed at 5 nm. Further optimization of the Al_2_O_3_ IL afforded a *V*_hy_ of 0.2 V, a mobility of 1.6 cm^2^ V^−1^ s^−1^, and an *I*_ON_/*I*_OFF_ of 1.2 × 10^5^.

In short, both SnO_2_ and SnO films made by ALD show good potential as semiconductor channel materials in FETs for promising applications in transparent and flexible electronics. The adaptability of ALD protocols also highlights its capacity to optimize tin oxide thin films for electronic applications. Finally, combining p‐type and n‐type tin oxides to construct p‐n junctions, inverters or other complex structures by ALD will also be a promising and significant direction toward advanced electronics in the future.

#### 2.1.4 Other Binary Metal Oxides

Semiconducting titanium oxide (TiO_2_) has also attracted extensive interest in the field of FETs due to its high transparency, good stability, and low‐cost growth process.^[^ ^82^, ^109^, ^125^ ^]^ Excellent electrical performance characteristics, such as a high field‐effect mobility of ≈10 cm^2^ V^−1^ s^−1^, attribute further to the use of TiO_2_ as a promising channel material.^[^ ^126^ ^]^

Early reports revealed that high‐quality TiO_2_ films fabricated by ALD can be applied as channel materials in FETs.^[^ ^82^, ^127^ ^]^ Ali et al. demonstrated TFT applications of TiO_2_ using thermal ALD in conjunction with post‐annealing.^[^ ^96^ ^]^ The annealed TiO_2_ film exhibited efficient electronic performance, with electron mobility of 0.672 cm^2^ V^−1^ s^−1^, an *I*_ON_/*I*_OFF_ of 2.5 × 10^6^, and a SS of 350 mV dec^−1^. Although TiO_2_ is typically considered as an n‐type semiconductor material owing to oxygen vacancies,^[^ ^109^ ^]^ p‐type behavior has also been demonstrated. Application of epitaxial growth mechanisms using ALD in combination with a [001] oriented Al_2_O_3_ substrate, can afford p‐type TiO_2_ (anatase) thin films.^[^ ^127^ ^]^ Furthermore, variation in post‐annealing conditions demonstrated that TiO_2_ may be natively p‐type and hole mobility may be further enhanced through titanium deficiencies.^[^ ^82^ ^]^

**Figure 8:**
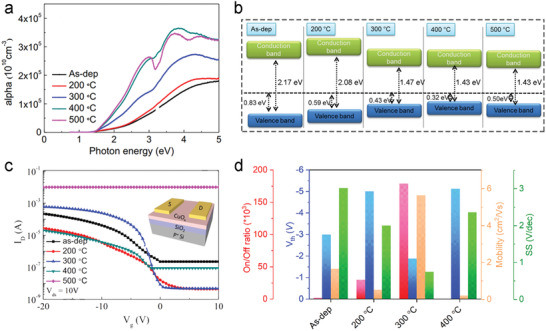
a) Tauc plot of the optical absorption and b) energy level diagram for the as‐deposited ALD‐CuO_x_ films and the annealed films at different temperatures. c) Transfer curves of TFTs based on the ALD‐CuO_x_ films. Reproduced with permission.^[^ ^94^ ^]^ Copyright 2016, Elsevier. d) Statistics of *μ*, *V*_T_, SS, and *I*_ON_/*I*_OFF_ depending on the annealing temperature, where these data were collected from the Ref. [94].

Copper oxide (CuO) and cuprous oxide (Cu_2_O), another family of promising binary oxide channel materials, likewise show p‐type behaviors and comparable electrical performance in electronic applications.^[^ ^94^, ^95^ ^]^ The influence of post‐annealing temperatures on the optical, electrical, and chemical properties of ALD‐CuO_x_ films deposited at 100 °C, were recently investigated by Maeng et al.^[^ ^94^ ^]^ Spectroscopic ellipsometry and X‐Ray photoelectron spectroscopy were used to distinguish the relationship between the optical bandgap and annealing temperature of the deposited films (**Figure** **8**a,b). The band edge position of ALD‐CuO_x_ films with controlled energy levels clearly relies on annealing temperatures. The as‐deposited ALD‐CuO_x_ film has an optical bandgap of ≈2.17 eV which then decreases to 2.08, 1.47, 1.43, and 1.43 eV according to the annealing temperatures of 200, 300, 400, and 500 °C, respectively. The electrical properties of the ALD‐CuO_x_ films were evaluated via TFT devices, revealing typical p‐type transfer characteristics (Figure 8c). The as‐deposited CuO_x_ exhibits a low *I*_ON_/*I*_OFF_ in addition to a high SS (Figure 8d), which may be attributed to poor stoichiometry at lower temperatures resulting in greater conducting behavior. The higher temperatures used during the annealing process enhance stoichiometry and thus crystallinity, leading to better semiconductor performance output. As a result, the optimized TFTs using ALD‐CuO_x_ film annealed at 300 °C showed overall improvement in electrical performance, such as the increased hole mobility and *I*_ON_/*I*_OFF_ of 5.64 cm^2^ V^−1^ s^−1^ and 10^5^, respectively. However, the formation of grain boundaries at 500 °C hinders the carrier transport, suppressing further enhancements in TFT performance achieved using post‐deposition annealing. Compared with typical p‐type CuO and Cu_2_O, some emerging metal halide semiconductors, such as p‐type cuprous halides (i.e., CuBr, CuI, and Zn‐doped CuI) and metal halide perovskites, can deliver better device performance of FETs.^[^ ^128^, ^129^, ^130^, ^131^, ^132^, ^133^, ^134^ ^]^ In particular, a very recent report by Liu and Noh et al. presented p‐channel perovskite FETs based on cesium tin triiodide (CsSnI_3_).The resultant FETs exhibited large field‐effect hole mobilities (>50 cm^2^ V^−1^ s^−1^), high *I*_ON_/*I*_OFF_ ratios of 10^8^, and excellent operational stability, demonstrating their promising potential for advanced electronics.^[^ ^134^ ^]^ Currently, there are several reports on the ALD of metal halides.^[^ ^135^, ^136^ ^]^ However, these ALD‐metal halides are isolated nanoparticles and evaluations of their semiconductor characteristics are not reported. Therefore, the research emphasis on high‐quality ALD p‐type semiconductors should not be limited to MOs. Metal halides and other inorganic hybrid materials are also promising avenues for exploration. The atomic layer control afforded by ALD is highly advantageous for the further development of conventional and emerging p‐type semiconductor channels and may yield new high‐performance transistors for electronic/optoelectronic applications.

#### 2.1.5 Multinary Metal Oxides

**Figure 9:**
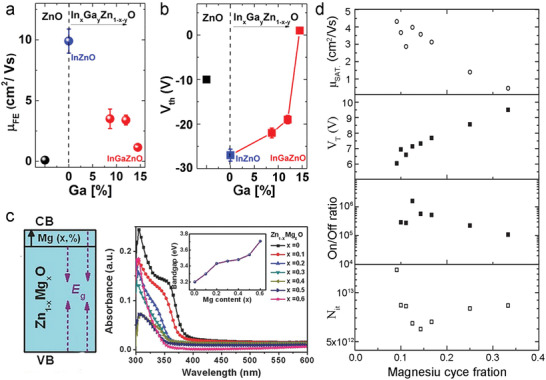
Trend in a) *μ* and b) *V*_T_ of TFTs based on ALD‐ZnO with different doped contents of Ga atom. Reproduced with permission.^[^ ^84^ ^]^ Copyright 2015, American Chemistry Society. c) Energy levels (left) and optical absorption spectra (right) of Zn_1‐x_Mg_x_O films with varied Mg contents. Reproduced with permission.^[^ ^107^ ^]^ Copyright 2014, Wiley‐VCH. d) Change in *µ*_sat_, *V*_T_, *I*_ON_/*I*_OFF_ and interface trap density (*N*_it_) of the ALD‐MZO TFTs depending on the Mg content. Reproduced with permission.^[^ ^141^ ^]^ Copyright 2014, AIP Publishing.

Multinary MOs such as IZO and amorphous indium gallium zinc oxide (*a*‐IGZO) are emerging as novel semiconductor materials for TFTs due to their flexible compositions and excellent electrical properties in contrast to conventional binary MOs.^[^ ^83^, ^84^ ^]^ The influence of elemental composition on the electrical properties of multi‐metal semiconductors has been well established within literatures.^[^ ^84^, ^85^ ^]^ For example, minor compositional alteration of *a*‐IGZO from In_0.45_Ga_0.15_Zn_0.40_O to In_0.38_Ga_0.18_Zn_0.44_O results in an 18% reduction in mobility from 48.3 to 39.4 cm^2^ V^−1^ s^−1^.^[^ ^85^ ^]^ In this respect, ALD affords facile tunability of metal precursor ratios; it enables the fabrication of multinary MOs in a precise manner. A unique approach accessible only using ALD applies the standard half‐reaction protocol, but includes an additional separate half‐reaction with a different metal precursor during the growth cycle.^[^ ^97^ ^]^ For example, alternating bilayers of In_2_O_3_ and ZnO were deposited according to a fixed ratio of 0.6 (6:4 half‐reactions per cycle, respectively), resulting in a pseudo‐multinary semiconducting heterostructure.^[^ ^137^ ^]^ Furthermore, selective deposition of the initial In_2_O_3_ layer onto the dielectric interface significantly improves TFT performance in comparison to a reverse ZnO‐first architecture. Indeed with field‐effect mobility (1.07 to 6.5 cm^2^ V^−1^ s^−1^), and *I*_ON_/*I*_OFF_ (5.2 × 10^6^ to 5.0 × 10^7^) are greatly improved whilst *V*_T_ (10 to 8.9 V) and SS (1.85 to 0.7 V dec^−1^) are deteriorated. In another study, Illiberi and coworkers deposited IZO using spatial‐ALD at atmospheric pressure.^[^ ^83^ ^]^ The ratio of indium/zinc (In/Zn) in the deposited film was accurately tuned by controlling the ratio of In/Zn precursor pulses. A 2:1 In/Zn ratio exhibits both high mobility (> 30 cm^2^ V^−1^ s^−1^) and good stability. The same team further designed quaternary zinc compounds of indium gallium zinc oxide with tunable electrical properties by adjusting the ALD cycle numbers of each binary MO.^[^ ^84^ ^]^ The field‐effect mobility of indium gallium zinc oxide decreased with an increase in Ga‐content, while the corresponding *V*_T_ rose significantly (**Figure** **9**a,b). The decreased mobility and increased *V*_T_ of oxide TFTs are attributed to the incorporation of Ga‐atoms in IZO, which suppresses oxygen vacancy formation through strong Ga‐O bond enthalpy.

It is worth noting that hetero‐element doping in binary MOs represents an effective strategy to produce several multinary MOs.^[^ ^98^ ^]^ For example, ZnO has been widely doped with aluminum, boron, indium, niobium, and magnesium, to improve its electrical properties and promote its applications in optoelectronic devices.^[^ ^64^, ^98^, ^138^, ^139^, ^140^ ^]^ Mg‐doped ZnO (MZO) films show gradual bandgap increases from 3.18 eV (binary ZnO) to 3.37 eV (ternary Zn_1‐x_Mg_x_O) after suitable Mg doping (Figure 9c).^[^ ^107^ ^]^ Nb‐dopants also exhibit analogous bandgap tunability when applied to ZnO films.^[^ ^98^ ^]^ Adaptation of precursor ratios during deposition cycles affords further compositional tuning possibilities for MZO films.^[^ ^141^ ^]^ Figure 9d exhibits the influence of Mg content in ALD‐MZO films on the TFT performance.^[^ ^141^ ^]^ With a cycle ratio of MgO/ZnO deposition increases from 1:10 to 1:2, the ALD‐MZO TFTs deliver suppressed electrical performance with a mobility decreased from 4.32 to 0.47 cm^2^ V^−1^ s^−1^ and a *V*_T_ increased from 6.05 to 9.51 V. In contrast, the *I*_ON_/*I*_OFF_ and *N*_it_ are almost unchanged.

**Figure 10:**
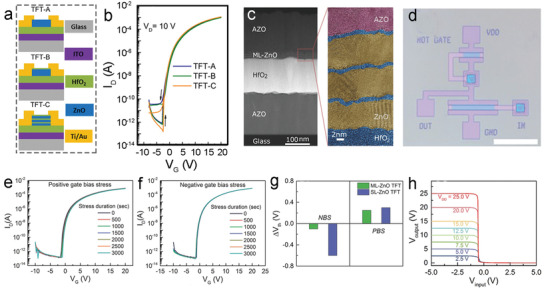
a) Schematic illustration of TFTs based on ALD‐ZnO/HfO_2_ with various structures and b) their corresponding transfer characteristics. Reproduced with permission.^[^ ^145^ ^]^ Copyright 2015, AIP Publishing. c) Cross‐sectional TEM image and false‐colored HRTEM image of the multinary‐ZnO film made by ALD. d) Optical image of a NMOS inverter based on the ALD ZnO‐based TFT (scale bar: 250 µm). Transfer curves of the TFT measured under e) PBS and f) NBS at room temperature. g) *V*_T_ shift for the SL‐ and ML‐ZnO TFTs was compared under PBS and NBS measurements. h) Voltage transfer curves of the NMOS inverter at different *V*_DD_ values. Reproduced with permission.^[^ ^146^ ^]^ Copyright 2016, Wiley‐VCH.

Among various doped‐ZnO materials, Al‐doped ZnO (AZO) thin films have received much attention owing to their unprecedented high mobility up to 136 cm^2^ V^−1^ s^−1^,^[^ ^142^ ^]^ as well as the low‐cost, high abundance, and non‐toxic nature of elemental Al.^[^ ^143^ ^]^ Investigation of Al‐doping effects on the bias‐stress stability of ALD‐AZO transistors indicates that 3% Al greatly preserves *V*_T_ hysteresis fidelity, whilst retaining adequate output curve characteristics.^[^ ^144^ ^]^ However, beyond 5% Al the formation of insulating Al_2_O_3_ greatly inhibits TFT performance. XRD analysis of the 3%‐AZO showed greatly improved grain orientation and size, which is postulated to enhance stability through the attenuation of trap site formation. Hf is also widely used as a doping element for achieving stable ZnO TFTs. Integration of novel channel architectures using n‐type Hf‐doped ZnO (HZO) has demonstrated improved bias stability, whilst retaining comparable mobility (4.2 cm^2^ V^−1^ s^−1^).^[^ ^102^ ^]^ The HZO/ZnO/HZO sandwich heterostructure affords enhanced surface layer stability under ambient conditions, whilst the additional HZO layer in contact with the dielectric interface reduces *V*_T_. Similarly, n‐type ZnO/HfO_2_ multilayer architecture has been fabricated as a channel in TFTs by ALD (**Figure** **10**a).^[^ ^145^ ^]^ This multilayer structure (TFT‐C) exhibits a comparable field‐effect electron mobility of ≈13.1 cm^2^ V^−1^ s^−1^, but a particularly high *I*_ON_/*I*_OFF_ of ≈8 × 10^9^ which is over 7 times higher than that of TFT‐A (pure ZnO) and TFT‐B (ZnO/HfO_2_) (Figure 10b). A multilayer structure analogous to that of TFT‐C was further investigated by HRTEM, as shown in Figure 10c.^[^ ^146^ ^]^ The multilayer heterostructure affords fully transparent devices and circuits, where all oxide components including the ZnO/HfO_2_ multilayer channel, AZO electrodes, and HfO_2_ dielectric were deposited by ALD and entirely indium‐free. The TFTs can be fabricated on polyethylene naphthalate (PEN) to deliver high electrical performance (*μ*_sat_ of 8.5 cm^2^ V^−1^ s^−1^, *I*_ON_/*I*_OFF_ over 10^9^, and SS value of 0.201 V dec^−1^). Moreover, the multilayer channel TFTs (ML‐TFTs) show a maximum *V*_T_ shift of only +0.3 V and −0.1 V after 3000 s of positive bias stress (PBS) and negative bias stress (NBS), respectively, demonstrating the excellent device operational stability (Figure 10e,f). Compared with the single‐layer TFTs (SL‐TFTs), the ML‐TFTs show a comparable *V*_T_ shift under the PBS but a much smaller *V*_T_ shift under the NBS (Figure 10g), due to efficient passivation of the multilayer ALD‐ZnO channel by the ultrathin HfO_2_ layer. The ML‐TFTs based on ALD‐ZnO can be further used for designing logic devices such as a negative channel MO semiconductor (NMOS) inverter (Figure 10d), displaying high‐performance static voltage transfer characteristics (Figure 10h). The voltage transfer curves exhibit the typical supply voltage (*V*_DD_) dependent rectangle shape. The output voltage (*V*_output_) remains the same with *V*_DD_ within the transition voltage (*V*_M_) and then drops immediately to *V*_GND_ when the input voltage (*V*_input_) exceeds *V*_M_.

In the interest of expanding the catalog of suitable materials for complementary logic circuits, research in p‐type MO semiconductors has increased in tandem in response to the rapid development of novel n‐type materials. The above examples highlight how sequential deposition using ALD may be used to fabricate high‐quality heterostructures. Thus, ALD remains an extremely promising technique for the development of future p‐n‐type heterojunctions.

### 2.2 Metal Oxide Insulators

Over the past decades, several MO insulators have been extensively utilized in electronic devices as gate dielectrics and/or protecting layers due to their high dielectric constant, large optical transparency, and excellent stability.^[^ ^87^, ^147^, ^148^, ^149^, ^150^ ^]^ Combining MO insulators with different semiconductor channel layers can significantly optimize the device performance and stability of FETs. Primarily, MO insulator/channel optimization has been demonstrated to greatly improve mobility, *I*_ON_/*I*_OFF_, hysteresis suppression, and operation stability.^[^ ^69^, ^151^ ^]^ It is worth noting that the trap state generated at the surface/interface of semiconductors such as those of group III‐V compounds are influenced by atmosphere or impurities, resulting in poor device performance.^[^ ^152^ ^]^ Thus, attenuation of trap density is necessary for improving the electrical performance of various FETs. Adoption of ALD‐processed MO insulators on group III‐V semiconductors suppresses interface trap state formation due to the high quality of oxide–dielectric or ‐passivation layer deposited.^[^ ^153^ ^]^ For instance, the ALD‐Al_2_O_3_/GaAs structure exhibited an upper limit for *N*_it_ of 5 × 10^11^–10^12^ cm^−2^eV^−1^.^[^ ^154^ ^]^ In this section, MO insulators fabricated by ALD are discussed. Influences on device performance, contribution as functional dielectrics, and protecting/encapsulating layers in FETs‐based electronic devices will be highlighted. Electrical performance parameters and transistor function of representative ALD‐MO insulators are summarized in **Table** **3**.

**Table 3:** Device performance of representative FETs with different MO insulators synthesized by ALD techniques

Material	Method	*T*_ALD_ [°C]	Precursors^a)^	Function & Device	Performance	Ref.
Al_2_O_3_	ALD	–	–	Interfacial passivation on p‐type SnO‐based TFTs	An improved *μ* from 1.8 to 5.4 cm^2^ V^−1^ s^−1^, a reduced SS from 0.88 to 0.51 V dec^−1^, and good stability in performance of the passivated SnO TFTs for over 1 year	[80]
	ALD	150	TMA + H_2_O	Organic/inorganic bilayer dielectric on OFETs	An improved *μ* from 0.08 to 0.65 cm^2^ V^−1^ s^−1^, a reduced leakage current, and an *I*_ON_/*I*_OFF_ of 1.6 × 10^4^	[147]
	PEALD	150	TMA + O_2_ plasma	Dielectric on WSe_2_‐based FETs	*μ* = 70.1 cm^2^ V^−1^ s^−1^, and *I*_ON_/*I*_OFF_ = 10^6^	[155]
	PEALD	250	TMA + H_2_O/O_2_ plasma	Dielectric on AlGaN/GaN MIS‐HEMTs	*V*_T_ = −5.8 V, SS = 68 mV dec^−1^, *V*_hy_ = 50 mV, and *I*_ON_/*I*_OFF_ = 10^10^	[149]
HfO_2_	ALD	150	TDMAHf + H_2_O	Dielectric on TMCs‐based TFTs	SS = 60 (MoS_2_) or 67 mV dec^−1^ (WSe_2_), and *I*_ON_/*I*_OFF_ = 10^7^ (MoS_2_ with < 5 nm HfO_2_)	[70]
	ALD	–	TEMAH + H_2_O	Dielectric and blocking oxide on MoS_2_‐based logic‐in‐memory devices	A memory window of 10.6 V, and a tunable *V*_T_ of memory devices by adding/removing charge carriers from the floating gate	[156]
	ALD	120	TDMAHf + H_2_O	Encapsulation on MoS_2_‐based FETs	Reduced hysteresis and retained on‐currents	[157]
BeO	ALD	250	DMBe + H_2_O	Dielectric on InGaAs‐based MOSFETs	A lower SS of ≈114 (BeO) than ≈120 (Al_2_O_3_) mV dec^−1^, and a higher *μ* of 1138 (BeO) than 847 (Al_2_O_3_) cm^2^ V^−1^ s^−1^	[158]
ZrO_2_	ALD	200	TDMAZr + H_2_O	Interlayer between the sapphire substrate and the *β*‐Ga_2_O_3_ channel	ZrO_2_ is beneficial for heat transfer from the channel to the sapphire substrate, resulting in a 35% less channel temperature increase	[159]
Y_2_O_5_	ALD	250	Y(MeCp)_3_ + H_2_O	Interlayer between the HfO_2_ dielectric and the native SiO_2_ layer (<1 nm)	A negative shift *V*_T_ of 224 mV; an improved *μ* from 228 (the single HfO_2_ dielectric) to 278 cm^2^ V^−1^ s^−1^ (with the Y_2_O_5_ interlayer)	[160]
La_2_O_3_	ALD	150	La(^i^PrCp)_3_ + H_2_O	Dielectric on InGaAs‐based MOSFETs	*SS* = ≈80 mV dec^−1^ for the devices with a 15 nm La_2_O_3_ dielectric	[161]
V_2_O_5_	ALD	50	V(dma)_4_ + H_2_O	Charge injection interlayer in OFETs	Three times higher *I*_DS_ of OFETs (with ≈1 nm VO_x_) than that of reference devices, and an improved *μ* from 0.29 to 0.80 cm^2^ V^−1^ s^−1^	[67]
TiAlO alloy	ALD	170	TMA + H_2_O TiCl_4_ + H_2_O	Dielectric on InGaAs/InP‐based MOSFETs	A breakdown field of 5.6 MV cm^−1^, low‐frequency dispersion (≈11%), and *V*_hy_ = 90 mV	[162]
Al_2_O_3_/Ta_2_O_5_	ALD	–	–	Bilayer dielectric on ZnO‐based TFTs	A low leakage current density of ≈10^−8^ A cm^−2^, an improved *μ* from 0.1 (single Ta_2_O_5_ dielectric) to 13.3 cm^2^ V^−1^ s^−1^ (bilayer Al_2_O_3_/Ta_2_O_5_ dielectric), and an *I*_ON_/*I*_OFF_ of 10^8^	[163]

^a)^ Note that TDMAHf is tetrakis(dimethylamino)hafnium; DMBe is dimethylberyllium; TDMAZr is tetrakis(dimethylamino)zirconium; Y(MeCp)_3_ is tris (methylcyclopentadienyl) yttrium; La(^i^PrCp)_3_ is tris(isopropylcyclopentadienyl)Lanthanum.

#### 2.2.1 Aluminum Oxide

Aluminum oxide (Al_2_O_3_) is widely applied as an insulator within electronic devices due to its high dielectric constant (*k*) and wide bandgap.^[^ ^164^, ^165^ ^]^ Currently, trimethylaluminum (TMA) is the most commonly used aluminum precursor for depositing Al_2_O_3_ by ALD.^[^ ^68^, ^150^, ^155^ ^]^ Utilizing an ALD‐Al_2_O_3_ dielectric, MgZnO‐based TFTs have shown improved electrical performance compared with devices with a SiO_2_ dielectric.^[^ ^166^ ^]^ The corresponding mobility and the *I*_ON_/*I*_OFF_ were improved from 5.65 to 7.73 cm^2^ V^−1^ s^−1^, and 4.4 × 10^5^ to 1.2 × 10^7^, respectively, while the SS was decreased from 0.80 to 0.29 V dec^−1^.

ALD‐Al_2_O_3_ has also been used as a gate dielectric layer in TMCs‐based FETs to improve their electrical performance.^[^ ^68^ ^]^ For instance, WSe_2_‐based FETs coupled with an ALD‐Al_2_O_3_ top‐gate dielectric layer exhibited excellent mobility of 70.1 cm^2^ V^−1^ s^−1^ and a high *I*_ON_/*I*_OFF_ of 10^6^.^[^ ^155^ ^]^ However, the lack of nucleation sites, which are mainly provided by dangling bonds on the surface of TMCs, hinders the formation of a conformal dielectric layer on the channel surface.^[^ ^167^ ^]^ Moreover, island‐like growth of high‐*κ* oxide clusters on the pristine MoS_2_ layer easily forms some defects such as pinholes, leading to increased gate leakage currents.^[^ ^70^, ^168^ ^]^ Therefore, thicker MO dielectrics are required for the top‐gated TMD transistors. High‐temperature requirements commonly observed for processing MO dielectrics may result in oxidation damage to TMC channels. Thus, deposition of high‐quality ultrathin MO dielectrics onto novel TMC channel materials remains a challenge for developing new TFT technologies.

**Figure 11:**
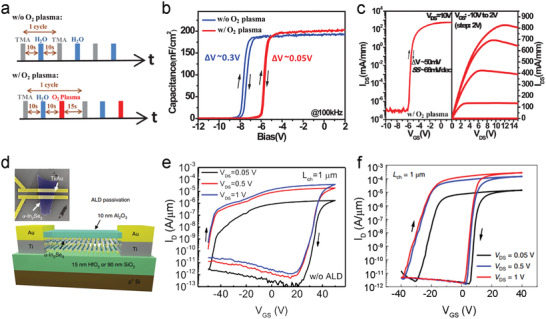
a) Schematic of the PEALD processes for depositing Al_2_O_3_ without (up) and with (down) O_2_ plasma. b) Capacitance‐bias characteristics of the GaN MIS‐diode with 10 nm Al_2_O_3_ films deposited without (blue) and with (red) O_2_ plasma. c) Transfer (left) and output (right) curves of GaN MIS‐HEMTs with the ALD‐Al_2_O_3_ top gate dielectric derived from O_2_ plasma. Reproduced with permission.^[^ ^149^ ^]^ Copyright 2018, IEEE. d) Top‐view SEM image (up) and schematic illustration (down) of the ALD‐Al_2_O_3_ passivated FET based on *α*‐In_2_Se_3_. Transfer curves of the *α*‐In_2_Se_3_ FET e) without and f) with the ALD‐Al_2_O_3_ passivation. Reproduced with permission.^[^ ^151^ ^]^ Copyright 2019, Nature Publishing group.

The interfacial chemistry of ReS_2_ was in situ analyzed with XPS during the deposition cycles of Al_2_O_3_.^[^ ^169^ ^]^ Standard PEALD combined with a UV‐Ozone pretreatment promotes the formation of weak S‐O bonds, which can facilitate nucleation and hence uniformity of the Al_2_O_3_ dielectric layer. Thus, the application of non‐thermal ALD techniques may be beneficial for suppressing non‐ideal surface growth mechanics among TMCs lacking appropriate nucleation density. The deposition of Al_2_O_3_ using TMA and water as co‐reactants has been widely adopted due to the well‐established robustness of the reaction and detailed mechanistic understanding.^[^ ^61^, ^68^, ^150^ ^]^ However, the stability of chemical bonds formed during this process, such as Al‐Al and Al‐O‐H, may lead to unwanted defects, which will influence the performance of final electronic devices.^[^ ^170^ ^]^ Previous literature has demonstrated that defect suppression occurs when using ozone (O_3_), through the removal of ‐OH groups,^[^ ^171^ ^]^ or direct use of O_3_ as a co‐reactant.^[^ ^172^ ^]^ The use of O_2_ plasma has also demonstrated reduced levels of ‐OH impurities, resulting in high‐quality Al_2_O_3_ films.^[^ ^173^ ^]^ Wang et al. deposited high‐quality Al_2_O_3_ gate dielectrics by PEALD using both H_2_O and O_2_ plasma as oxygen sources within one cycle (**Figure** **11**a down).^[^ ^149^ ^]^ Compared with a sample without O_2_ plasma (Figure 11a above), AlGaN/GaN‐based metal‐insulator‐semiconductor high electron mobility transistors (MIS‐HEMTs) with the O_2_ plasma‐derived Al_2_O_3_ dielectric exhibit improved electrical performance with a much smaller hysteresis (Figure 11b). Moreover, a lower SS of 68 mV dec^−1^ and a higher *I*_ON_/*I*_OFF_ of ≈10^10^ are achieved compared with a SS of 97 mV dec^−1^ and an *I*_ON_/*I*_OFF_ of ≈10^8^ for the sample without O_2_ plasma (Figure 11c).

As discussed above, besides the trap states induced by —OH impurities in the Al_2_O_3_ dielectric, surface oxides on the surface of non‐oxide semiconductors, particularly for group III‐V semiconductors, seriously degrade the electrical performance of final devices.^[^ ^174^, ^175^ ^]^ Indeed, eliminating surface oxidization of the semiconductor and reducing the interfacial trap state density are effective strategies to improve the electrical performance of group III‐V semiconductor‐based MOSFETs.^[^ ^175^, ^176^ ^]^ Native oxide ILs on group III‐V semiconductors are diminished during Al_2_O_3_ deposition by ALD due to the formation of volatile IL products and conversion of interfacial oxides to Al_2_O_3_. The so‐called interfacial oxide self‐cleaning, resulted in lower trap densities and better electrical properties.^[^ ^152^, ^177^, ^178^ ^]^ Moreover, the ALD‐Al_2_O_3_ dielectric as an encapsulation layer efficiently minimized the moisture‐absorption effects on the insulator/semiconductor interface, thus contributing to a more stable interface state and improved electrical performance.^[^ ^179^ ^]^

High‐temperature induced surface oxidization of the semiconductors whilst depositing high‐*k* oxides by ALD is another factor that leads to the degradation of electrical performance for FET‐based devices. Therefore, decreasing the ALD temperature for processing MO insulators is an attractive route to improve device performance.^[^ ^180^ ^]^ Decreased temperature during ALD of an Al_2_O_3_ dielectric layer onto p‐type GaSb‐based FETs has resulted in an increase in the hole mobility.^[^ ^176^ ^]^ This improvement may be attributed to suppressed oxidization and trap state density at the semiconductor/insulator interface, which is known to easily occur in response to higher ALD temperatures. As a result, compared with the high‐temperature derived Al_2_O_3_, GaSb‐based MOSFETs with the ALD‐Al_2_O_3_ dielectric deposited at only 150 °C delivered a highly decreased *N*_it_ of ≈4.5 × 10^13^ cm^−2^ eV^−1^. Enhanced TFT performance at lower ALD temperatures was also reflected in the deposition of an Al_2_O_3_ dielectric/passivation layer on a polycrystalline diamond. Devices fabricated at 200 °C displayed a higher output current, larger *V*_T_, and lower on‐resistance compared with that deposited at 300 °C.^[^ ^180^ ^]^

In addition, ALD‐processed Al_2_O_3_ has been also employed as an effective passivation layer to reduce the surface trap density, and thus suppress the threshold voltage hysteresis significantly.^[^ ^80^ ^]^ Si et al. demonstrated enhanced FET performances through the passivation of indium selenide (*α*‐In_2_Se_3_) by ALD‐Al_2_O_3_.^[^ ^151^ ^]^ Figure 11d presents a top‐view SEM image (up) and a schematic device (down) of *α*‐In_2_Se_3_‐FETs with the ALD‐Al_2_O_3_ passivation. Non‐passivated FETs exhibit transfer curves with clockwise hysteresis loops with large memory windows (>70 V) at different *V*_DS_ values (Figure 11e), whilst *I*_ON_/*I*_OFF_ remains above 10^7^ at a *V*_DS_ of 0.5 V. By contrast, the ALD‐Al_2_O_3_ passivated FETs exhibit clockwise hysteresis loops with reduced memory windows and an enhanced *I*_ON_/*I*_OFF_ of 10^8^ at a *V*_DS_ of 1 V, as well as a low SS of 0.65 V dec^−1^ and a significantly improved mobility of 312 and 488 cm^2^ V^−1^ s^−1^ in the forward and reverse sweep, respectively (Figure 11f).

Additionally, the adoption of an ALD‐Al_2_O_3_ passivation interlayer between the p‐type SnO film and the SiO_2_ dielectric layer can efficiently improve the mobility of SnO TFTs from 1.8 to 5.4 cm^2^ V^−1^ s^−1^, whilst decreasing the *V*_hy_ from 4 to 0.2 V.^[^ ^80^ ^]^ The passivated SnO channel showed outstanding electrical performance stability and negligible degradation after long‐term storage for over 1 year. Enhanced performance of OFETs was also observed when inserting a thin ALD‐Al_2_O_3_ interlayer between the organic dielectric and semiconductor layers.^[^ ^147^ ^]^ Passivated OFETs with the ALD‐Al_2_O_3_ dielectric interlayer (only 1 nm) showed a significantly reduced leakage current and elevated mobility of 0.65 cm^2^ V^−1^ s^−1^, much higher than 0.08 cm^2^ V^−1^ s^−1^ for the devices without an Al_2_O_3_ interlayer.

In short, ALD is a mature technique to grow Al_2_O_3_ films for electronic applications that can participate in the improvement of device performance. However, the film quality (i.e., film thickness, surface roughness, and pinhole defects) is still one of the main limits that hinder the progress of ALD‐Al_2_O_3_ towards high‐performance electronic devices. In addition, the corrosion of ALD‐Al_2_O_3_ films in a humid environment remains a problem requiring a solution.^[^ ^181^ ^]^

#### 2.2.2 Hafnium Dioxide

Hafnium dioxide (HfO_2_) is an insulating MO with a bandgap of 5.3–5.7 eV.^[^ ^76^, ^182^ ^]^ Since 2007, a hafnium‐based oxide was introduced to replace conventional SiO_2_ in FETs by Intel, since then it has become a widely used high‐*k* gate dielectric due to its suitable band offset with Si and excellent dielectric constant of 25, far higher than that of SiO_2_.^[^ ^183^ ^]^

In relation to the synthesis of hafnium‐based oxides, ALD is one of the most promising techniques to design high‐quality HfO_2_ films with controllable thickness and high conformality. Various hafnium‐based precursors such as halides and amides have been utilized for preparing ALD‐HfO_2_ films.^[^ ^70^, ^157^, ^183^ ^]^ As an alternative to the normally used oxygen source (e.g., water, O_2_ and O_3_), carboxylic acids have also been demonstrated for the ALD of high‐quality HfO_2_ films.^[^ ^184^, ^185^, ^186^ ^]^ However, a point of concern is the incorporation of inorganic/organic ligands in the deposited films during the ALD process significantly affects their final electrical properties.^[^ ^183^ ^]^ Therefore, it is particularly important to select suitable metal precursors and oxygen sources for developing high‐quality HfO_2_ films with excellent properties towards electronic applications.

Among the hafnium‐based precursors, hafnium tetrachloride (HfCl_4_) is commonly used for depositing HfO_2_ dielectric films due to its good thermal stability, which ensures the minimized decomposition of metal precursors.^[^ ^183^, ^187^, ^188^ ^]^ HfO_2_ films deposited with HfCl_4_ also show greater stoichiometry, crystallinity, and lower leakage currents in Si‐based electronic devices compared with those derived from TDMAHf.^[^ ^183^ ^]^ In addition, HfO_2_ films made from HfCl_4_ display improved electrical performance in graphene‐based FETs due to better nucleation of HfCl_4_ on 2D graphene substrates. It should be noted that HCl is the by‐product generated when using HfCl_4_ and may cause etching damage and hence hinder device performance.

In addition to metal precursors, oxygen source selection is a key parameter that can affect the electrical performance of electronic devices. Compared with a water‐derived HfO_2_ film, the ozone‐derived HfO_2_ layer has given rise to higher threshold voltages and lower gate leakage currents in the metal‐oxide semiconductor heterostructure FETs (MOS‐HFETs).^[^ ^189^ ^]^ Post‐deposition annealing is another factor that can be tuned to optimize both structural characteristics and electrical properties of the ALD‐HfO_2_ films.^[^ ^190^ ^]^

**Figure 12:**
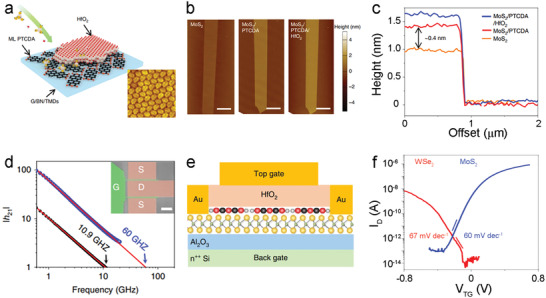
a) Schematic of ALD‐HfO_2_ stacked on 2D materials with a ML PTCDA interlayer. The inset is a high‐resolution scanning tunneling microscope (HR‐STM) image of the ML PTCDA on graphene with 10 × 10 nm^2^ scale. b) AFM images and c) Height profile of exfoliated MoS_2_ (left), deposited with ML PTCDA (middle) and after 2 nm ALD‐HfO_2_ (right). d) Small signal current gain |h_21_| for graphene‐based FETs before (black symbols) and after (blue symbols) de‐embedding, measured under *V*_DS_ = 1 V and *V*_G_ = −0.4 V. e) Schematic structure of the TMC‐TFTs with a ML PTCDA interlayer and an ALD‐HfO_2_ dielectric. f) Transfer curves of FETs based on ML MoS_2_ (blue) and WSe_2_ (red) with the 3 nm ALD‐HfO_2_. Reproduced with permission.^[^ ^70^ ^]^ Copyright 2019, Nature Publishing group.

Recently, the incorporation of HfO_2_ into 2D semiconductor devices has also emerged as a promising development for enhanced device performance. However, homogenous nucleation of ultrathin dielectric films remains a significant challenge during the ALD process. Price et al. demonstrated the deposition of a sub‐5 nm uniform HfO_2_ dielectric onto 2D MoS_2_ and WSe_2_ using PEALD when fabricating top‐gate FETs.^[^ ^191^ ^]^ Compared with thermal ALD, PEALD can yield significantly improved nucleation on 2D crystals, resulting in a uniform and smooth HfO_2_ thin‐films. Plasma damage to the surface of TMCs remains an issue when processing 2D materials, which may limit its application for 2D TMCs approaching monolayer thickness.^[^ ^157^ ^]^ Similar to the utilization of perylene tetracarboxylic acid (PTCA) for depositing uniform Al_2_O_3_ on graphene,^[^ ^192^ ^]^ a monolayer (ML) molecular crystal of 3,4,9,10‐perylene‐tetracarboxylic dianhydride (PTCDA) was used as a nucleation layer to deposit ultrathin HfO_2_ dielectrics by ALD on 2D materials including graphene, MoS_2_, and WSe_2_ (**Figure** **12**a).^[^ ^70^ ^]^ This method affords reduced density of trap states at the interface, suppressed leakage currents, and an improved breakdown field. AFM images (Figure 12b) and height profiles (Figure 12c) support the uniform deposition of both PTCDA ML film and the HfO_2_ dielectric layer. Graphene‐based FETs with the hybrid PTCDA/ALD‐HfO_2_ dielectric show a high mobility (≈3500 cm^2^ V^−1^ s^−1^), and outperform an intrinsic cutoff frequency (ƒ_T_) reaching 60 GHz (Figure 12d). Integration of ML‐PTCDA/ALD‐HfO_2_ (3 nm) within monolayer TMC FETs (Figure 12e) brings excellent transfer characteristics (Figure 12f). Both MoS_2_ and WSe_2_ FETs respectively displayed *I*_ON_/*I*_OFF_ ratios of 10^7^ and 10^6^, with SS values of 60 and 67 mV dec^−1^.

Passivation of 2D TMC interfaces is another feature of ALD‐HfO_2_, which may be exploited for improving device performance.^[^ ^69^, ^193^ ^]^ TMC semiconductors are sensitive to surface oxidation reactions from atmospheric moisture and oxygen, leading to degradation and poor device longevity. Therefore, passivation and encapsulation using high‐*k* dielectric materials are required to develop high‐performance TMC transistors.^[^ ^69^, ^77^ ^]^ For example, the hysteresis of HfS_2_ transistors reduced significantly after passivation using ALD‐HfO_2_ and corresponded with enhancement to the source‐drain current.^[^ ^188^ ^]^ FETs using a MoS_2_ channel encapsulated by an ALD‐HfO_2_ layer can also display enhanced electrical performance compared with reference devices without encapsulation.^[^ ^69^, ^168^ ^]^ Layer thickness of HfO_2_ may also reduce charge impurity‐induced scattering, in addition to screening from atmospheric interferences.^[^ ^168^ ^]^

**Figure 13:**
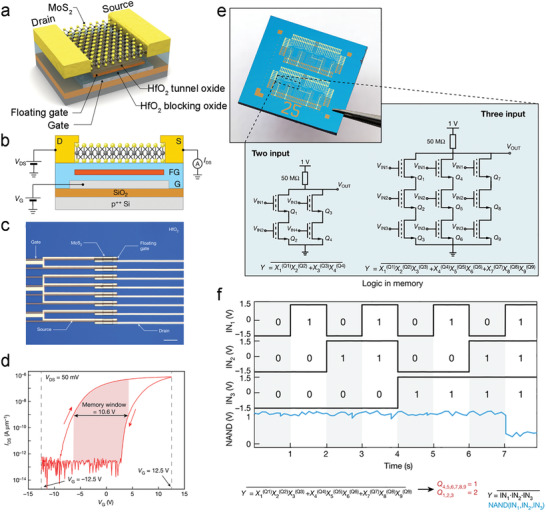
a) 3D view and b) cross‐section view of a floating‐gate MoS_2_‐based memory device with ALD‐HfO_2_ as a blocking and dielectric oxide. c) Optical image of the MoS_2_‐based floating‐gate memory array with the ALD‐HfO_2_. d) Transfer curves of the FGFET with a wide memory window of 10.6 V. e) Schematic of two‐input and three‐input logic‐in‐memory devices (boxed area) and photo of a fabricated 12 × 12 mm^2^ die with corresponding devices arrays (top left). f) Output voltages of the three‐input device with the NAND logic operation, indicating a stabile operation. Reproduced with permission.^[^ ^156^ ^]^ Copyright 2020, Nature Publishing group.

More practical electronic device application of ALD‐HfO_2_, was demonstrated by Marega et al. through designing floating‐gate FETs (FGFETs) for logic‐in‐memory devices and circuits (**Figure** **13**a–c).^[^ ^156^ ^]^ The ALD‐HfO_2_ dielectric film can work as a blocking and tunnel oxide to efficiently modulate the electric field within the MoS_2_ channel. Basic characterization of the devices is performed under a *V*_DS_ of 50 mV and a *V*_G_ ranging from −12.5 to 12.5 V, delivering a memory window of 10.6 V (Figure 13d). Moreover, the FGFETs are further designed and integrated into two‐input and three‐input logic circuits (Figure 13e). As a representative, a complete three‐input NAND logic, is normally operated and its corresponding logic input and output curves are shown in Figure 13f. The stable operation of these reprogrammable logic‐in‐memory devices highlights the practical benefits of adopting ALD for optimizing FET performance in developing areas of the electronics industry.

#### 2.2.3 Other Insulating Metal Oxides

Despite rapid advances in the commonly used Al_2_O_3_ and HfO_2_, additional insulating MOs such as ZrO_2_,^[^ ^194^ ^]^ BeO,^[^ ^158^ ^]^ La_2_O_3_,^[^ ^161^ ^]^ and Y_2_O_5_^[^ ^160^ ^]^ have recently attracted attention in electronics owing to their high dielectric constants and large bandgap values. For example, Anderson et al. deposited a ZrO_2_ gate dielectric on AlGaN/GaN MOSFETs by ALD, resulting in a much lower interface trap site at a density of 7.00 × 10^12^ cm^−2^eV^−1^ than that of the Schottky‐gate reference device (2.03 × 10^13^ cm^−2^ eV^−1^).^[^ ^195^ ^]^ Moreover, the gate leakage current was reduced by up to four orders of magnitude. Chen and co‐workers encapsulated organic light‐emitting diodes (OLEDs) with ZrO_2_ by PEALD and molecular layer deposition (MLD), leading to the efficiently improved water barrier properties of up to 3.08 × 10^−5^ g m^−2^ day^−1^.^[^ ^196^ ^]^ In addition, multinary ALD‐MO insulators such as titanium aluminum oxides (TiAlO),^[^ ^162^ ^]^ and magnesium calcium oxides (MgCaO),^[^ ^197^ ^]^ are also promising candidates as gate dielectrics or encapsulating materials.

The potential size reduction obtained from using thinner MO with high dielectric constants, such as ZrO_2_ and Ta_2_O_5_, make ALD a promising technique for novel device manufacturing.^[^ ^198^ ^]^

#### 2.2.4 Metal Oxide Dielectric Heterostructures

Insulating single MO dielectrics have been successfully applied on various channel layers to improve the electrical performance of the final devices. However, it is still a challenge to deposit ultrathin, continuous, and pinhole‐free MO films on the surface of a channel layer due to nucleation site deficiency, especially on 2D channel materials.^[^ ^68^, ^70^ ^]^ Therefore, several methods have been employed to promote surface nucleation and obtain high‐quality dielectric layers. Examples include, pretreatment (activation) of the substrate surface before the ALD reaction, using strong oxidants like O_3_ or O_2_ plasma during the ALD process, and adopting higher reaction temperatures.^[^ ^149^, ^171^ ^]^ As discussed in previous sections (ALD‐Al_2_O_3_ and ALD‐HfO_2_), the oxidization of channel materials is predominantly caused by high deposition temperatures and the use of strong oxidizing agents. This remains a consistent hindrance to the broad adoption of ALD in the fabrication of ultrathin MO dielectric films.^[^ ^149^, ^163^, ^199^ ^]^

A successful strategy to suppress channel layer degradation during ALD and enhance dielectric properties is the incorporation of an intermediate layer to design MO dielectric heterostructures.^[^ ^163^, ^199^, ^200^ ^]^ Similar to the use of a PTCDA seeding layer as mentioned above, an ultrathin MO layer is preemptively deposited onto the channel as a seeding layer prior to MO ALD. For example, an Al_2_O_3_/ZrO_2_ dielectric heterostructure was deposited onto MoS_2_, yielding significant improvements to device performance.^[^ ^199^ ^]^ A 5 nm layer of Al_2_O_3_ was initially deposited onto the MoS_2_ channel by ALD at 150 °C using TMA and H_2_O as precursors. The corresponding second ZrO_2_ dielectric (45 nm) was then deposited at an increased temperature (300 °C) onto the Al_2_O_3_ seeding layer. Compared with the MoS_2_‐TFTs using a single ZrO_2_ dielectric layer, the TFTs decorated with the Al_2_O_3_/ZrO_2_ heterostructure dielectric show better electrical performance with improved mobility from 5.1 to 7.1 cm^2^ V^−1^ s^−1^.

**Figure 14:**
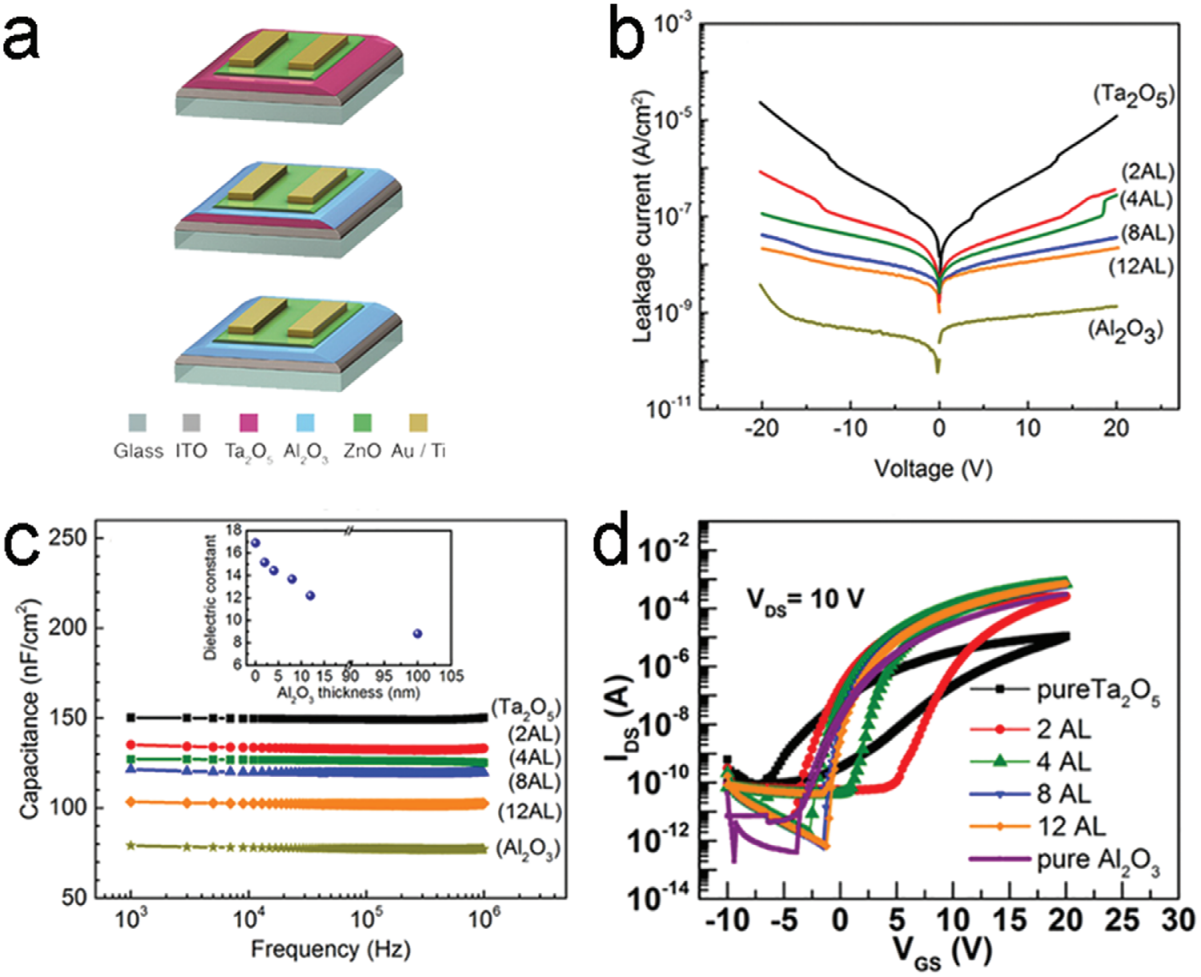
a) Schematic of ZnO‐TFTs with a single Ta_2_O_5_ dielectric layer (up), a heterostructured ALD‐Al_2_O_3_/Ta_2_O_5_ dielectric (middle) and a single Al_2_O_3_ dielectric layer (down). b) Leakage current density–voltage characteristics and c) capacitance–frequency curves of different ALD‐MO dielectrics measured in metal/dielectric/metal capacitors. The inset of c) indicates the dielectric constant varied with the thickness of Al_2_O_3_ interlayer. d) Transfer curves of ZnO‐based TFTs with the single and bilayer ALD‐MO dielectrics. Reproduced with permission.^[^ ^163^ ^]^ Copyright 2016, American Chemical Society.

Besides deposition of high‐quality dielectric films onto a channel layer, an intermediate/nucleation layer in heterostructured MO dielectrics can also protect channel materials during the ALD process, and prevent the creation of metal atomic vacancies or interstitial defects.^[^ ^68^, ^163^ ^]^ For example, the formation of Zn vacancies during the ALD of a ZnO film onto a dielectric Ta_2_O_5_ layer leads to inferior properties of ZnO TFTs.^[^ ^163^ ^]^ To solve this problem, a thin ALD‐Al_2_O_3_ layer has been adopted between the ZnO channel layer and the Ta_2_O_5_ dielectric layer to improve the electrical performance of ZnO‐based TFTs (**Figure** **14**a).^[^ ^163^ ^]^ Compared with a single Ta_2_O_5_ dielectric, a series of Al_2_O_3_/Ta_2_O_5_ dielectric heterostructures grown by ALD show comparable dielectric constants but dramatically suppressed leakage currents (Figure 14b). Further increasing the Al_2_O_3_ film thickness results in the gradual attenuation of leakage current density, which is beneficial for reducing off‐currents and improving total device performance. These ALD‐MO dielectrics also have good stability in capacitance at frequencies from 1 kHz to 1 MHz (Figure 14c). As a result, the ZnO‐TFTs with an optimized Al_2_O_3_/Ta_2_O_5_ dielectric show a greatly improved mobility (13.3 cm^2^ V^−1^ s^−1^) than that of the single Ta_2_O_5_ dielectric layer (0.1 cm^2^ V^−1^ s^−1^), as well as a suppressed SS value and an improved *I*_ON_/*I*_OFF_ (Figure 14d).

Examples of advanced dielectric heterostructures using sputtering deposition (SD) of Ta_2_O_5_ onto an ALD‐Al_2_O_3_ thin buffer layer have also been demonstrated.^[^ ^201^ ^]^ In contrast with the SD method, ALD techniques have several additional advantages for depositing MO dielectrics, including low growth temperatures, uniform film deposition, and controllable film thicknesses. Moreover, transfer from ALD to SD chamber may introduce surface‐adsorbed air contaminants, which may impact the subsequent purity of SD‐deposited films and hence the electronic performance of TFTs. Liu et al. studied the electrical properties of different TiO_2_/Al_2_O_3_ dielectrics which were prepared by SD and ALD, respectively.^[^ ^202^ ^]^ Compared with MOSFETs with the SD‐TiO_2_/Al_2_O_3_ dielectric, the devices with the ALD‐TiO_2_/Al_2_O_3_ films exhibit a much lower leakage current density and better capacitance‐voltage characteristics due to the better quality of thin‐film MO dielectric heterostructures made by ALD.

Apart from utilizations in inorganic semiconductors‐based FETs, MO dielectric heterostructures have also been widely used in OFETs for enhancing their electrical performance.^[^ ^181^, ^200^ ^]^ Composition and thickness are important parameters of MO dielectrics and strongly influence transistor performance.^[^ ^163^, ^203^, ^204^ ^]^ Thus, MO heterostructures with suitable compositions and optimized thicknesses for TFTs remain a major research focus for ALD.

### 2.3 Metal Oxide Electrodes and Electrode Interlayers

**Figure 15:**
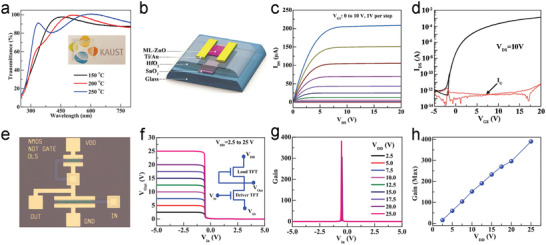
a) UV–vis spectra of ALD‐SnO_2_ films deposited at varied temperatures. b) Schematic structure of the SnO_2_‐gated TFT. c) Output and d) transfer curves of the TFT with the ALD‐SnO_2_ gate electrode deposited at 200 °C. e) Optical image of a NMOS inverter based on the ALD‐SnO_2_ gate. f) Voltage transfer curves of the inverters with a schematic circuit as shown in the inset, g) Voltage gain curves at different *V*_DD_ values and h) the corresponding statistical result. Reproduced with permission.^[^ ^66^ ^]^ Copyright 2017, Wiley‐VCH.

In addition to its application as semiconductor channel materials and insulators, ALD‐MOs have been adopted for use as transparent conductive oxide electrodes (TCO) and semiconducting electrode interlayers in transistors. For example, indium‐tin‐oxide (ITO), a well‐known heteroatom doped SnO_2_, is widely employed as a kind of TCO electrode.^[^ ^205^ ^]^ Introduction of heteroatoms into oxides increases the carrier concentration, thus converting MO semiconductors to conductors. For pure SnO_2_ made by ALD, it can be transformed from the semiconductor to conductor by adjusting ALD conditions (i.e., temperature) to alter carrier concentrations.^[^ ^92^ ^]^ For instance, highly conductive SnO_2_ serving as a transparent gate electrode has been fabricated by controlling the ALD reaction temperature.^[^ ^66^ ^]^ The ALD‐SnO_2_ films deposited at 200 °C exhibit a large carrier concentration (>10^20^ cm^−3^), a low resistivity (≈0.0031 Ω cm), and a high transparency (≈93%) in most of the visible range (**Figure** **15**a). ZnO‐based TFTs with such an ALD‐SnO_2_ gate electrode (Figure 15b) exhibit excellent electrical performance with a high saturation mobility of 15.3 cm^2^ V^−1^ s^−1^, a low SS of 130 mV dec^−1^, a very high *I*_ON_/*I*_OFF_ ratio of ≈10^9^, and a low gate leakage current (*I*_G_) of less than 10^−12^ A (Figure 15c,d). The ALD‐SnO_2_ gated‐TFTs outperform other devices based on electrodes of conventional ITO (*μ* = 15 cm^2^ V^−1^ s^−1^, SS = 160 mV dec^−1^, and *I*_on_/*I*_off_≈10^9^) and ALD‐AZO (*μ* = 11 cm^2^ V^−1^ s^−1^, SS = 180 mV dec^−1^, and *I*_on_/*I*_off_≈10^9^). Based on these findings, NMOS inverters were designed to further demonstrate the practical application of ALD‐SnO_2_ gate electrodes. The NMOS inverter with the ALD‐SnO_2_ gate (Figure 15e) displays static voltage‐transfer curves (Figure 15f), highlighting its excellent room temperature performance under varying *V*_DD_ values ranging from 2.5 to 25 V. Moreover, the maximum gain values of the inverter gradually increase from 20 to 382 upon raising *V*_DD_ from 2.5 to 25 V (Figure 15g,h).

Additionally, transition metal oxides (TMOs) such as VO_x_,^[^ ^67^ ^]^ and MoO_3_,^[^ ^206^ ^]^ possess suitable semiconducting properties and high stability, enabling them to serve as electrode interlayers for efficient charge injection in optoelectronic/electronic devices.^[^ ^207^ ^]^ Large contact resistance at organic/metal interfaces seriously limits the carrier mobility and *I*_ON_/*I*_OFF_ of OFETs.^[^ ^208^ ^]^ Reduction of interfacial contact resistance has been demonstrated using an ultrathin ALD‐VO_x_ film at the electrode/semiconductor interface in OFETs. It functions as a hole injection layer, promoting electrode work function tunability and charge transport.^[^ ^67^ ^]^ The VO_x_ interlayer with controlled thicknesses was deposited by ALD with tetrakis(dimethylamino) vanadium [V(dma)_4_] and H_2_O at a low temperature (50 °C). After 20 ALD cycles, the resulting OFETs exhibited remarkably improved electrical performance with a reduced contact resistance from 71 to 10 kΩ cm and enhanced mobility from 1.09 to 1.56 cm^2^ V^−1^ s^−1^. The ALD‐VO_x_ interlayer also performed well as a barrier material against moisture/oxygen transmission in OFETs, delivering a good retention (over 83% after 30 days) in the mobility for devices with a combined ALD‐Al_2_O_3_ layer for encapsulation.

## 3 ALD of Metal Chalcogenides for FETs

Interest in applications of MCs and particularly TMCs within next‐generation electronics has risen steeply over the past decade, driven by their intrinsic layered crystal structures and unique properties.^[^ ^193^, ^209^ ^]^ TMCs typically consist of a metal/chalcogen atomic ratio of 1:2, although exceptions of 1:1,^[^ ^210^, ^211^ ^]^ and 2:3^[^ ^212^ ^]^ are possible. TMCs have three classical polymorphs, which are tetragonal (1T), hexagonal symmetry (2H), and rhombohedral (3R).^[^ ^213^ ^]^ Their electrical properties strongly depend on the polymorphs and structural phase transition, where the TMCs with 1T and 2H phases show metallic and semiconducting behaviors, respectively. For further details on TMCs, their chemical compositions, electronic structures, and applications have been described in a comprehensive review by Chhowalla and co‐workers.^[^ ^214^ ^]^

Versatile processing methods such as exfoliation,^[^ ^215^ ^]^ CVD,^[^ ^216^ ^]^ and magnetron sputtering^[^ ^217^ ^]^ have been reported for the synthesis of TMCs for applications in electronics. However, intrinsic factors arising from these methods often limit the fabrication of TMC films in a uniform, repeatable, scalable, and tunable manner. Precise film thickness is of particular importance to TMCs due to their layer‐dependent bandgap characteristics. Bandgap magnitude and crystal structures are influenced by the thickness of TMCs, resulting in a transition from an indirect to direct bandgap material, in the bilayer to a monolayer transition.^[^ ^213^ ^]^

The tunability of ALD has established it as an ideal candidate for the fabrication of high‐quality MCs as channel materials, particularly with regard to thickness control at the nanoscale.^[^ ^218^, ^219^ ^]^ Channel length (*λ*) is affected by the thickness of both channel and dielectric layers according to the following Equation (4),

(4) $$\lambda =\sqrt{\frac{\varepsilon_{\mathrm{ch}}}{N\varepsilon_{\mathrm{ox}}}d_{\mathrm{ch}}d_{\mathrm{ox}}}$$

where *N* is the number of gates, and the *ε*_ch_ (*d*_ch_) and *ε*_ox_ (*d*_ox_) are the dielectric constants (thickness) of the channel and the oxide, respectively.^[^ ^220^ ^]^ Thus, ALD is an efficient technique to minimize the size of electronic devices by using ultrathin channel materials, such as few‐ or mono‐layer (<1 nm) TMCs.^[^ ^198^ ^]^

This section will discuss MCs and their heterostructures fabricated using ALD for applications in transistors, with an emphasis on film structural characteristics and device performances. Representative examples of various MCs synthesized by ALD and their related electrical properties as well as device performance are presented in **Table** **4**.

**Table 4:** Device characteristics of representative FETs with semiconducting MCs synthesized by ALD techniques

Material	Method	*T*_ALD_	Precursors^a)^	Mobility [cm^2^ V^−1^ s^−1^]		*I*_ON_/*I*_OFF_	SS	Ref
		[°C]		n‐type	p‐type		[V dec^−1^]	
MoS_2_	ALD	60	Mo(NMe_2_)_4_ + H_2_S	0.23	–	10^2^	–	[73]
	ALD	350–450	MoCl_5_ + H_2_S	1	–	–	–	[221]
	ALD	700	MoF_6_ + H_2_S MoCl_5_ + H_2_S	0.1	–	10^6^	3.5	[222]
	ALD	300	MoCl_5_ + HMDST	≈3	–	–	–	[223]
	ALD	400	MoCl_5_ + HMDST	0.02	–	>10^2^	–	[224]
	ALD	250	Mo(CO)_6_ + DEDS	13.9	–	>10^8^	–	[209]
	SLS	500–900	MoCl_5_ + H_2_S	0.2	–	10^8^	0.36	[225]
	Sulfurizing ALD‐MoO_3_	155	Mo(CO)_6_ +O_2_ plasma	5.9	–	≈10^4^	–	[219]
WS_2_	ALD	390	WCl_5_ + H_2_S	12	–	>10^4^	–	[226]
	PEALD	300–450	WF_6_ + H_2_ plasma + H_2_S	–	–	10^5^	–	[227]
	Sulfurizing ALD‐WO_3_	300	WH_2_(^i^PrCp)_2_ + O_2_ plasma	3.9	–	–	0.6	[228]
	Sulfurizing PEALD‐WO_3_	180	BTBMW + O_2_ plasma	4.5	–	10^5^	–	[229]
WSe_2_	ALD	390	WCl_5_ + H_2_Se	633	350	10^5^	–	[230]
	SLS	600–800	WCl_6_ + DESe	–	2.2	10^6^	–	[231]
SnS_2_	ALD	150	TDMASn + H_2_S	≈0.079	–	8.3 × 10^6^	0.83	[232]
	ALD	150–240	Sn(dmamp)_2_ + H_2_S plasma	0.8	–	10^6^	–	[74]
	Sulfurizing ALD‐SnO_x_	350	Sn(dmamp)_2_ + H_2_O	0.02	–	10^5^	–	[233]
SnS	ALD	170	TDMASn + H_2_S	–	0.21	8.8	–	[234]
	ALD	120	Sn(amd)_2_ + H_2_S	–	15.3^b)^	–	–	[235]
	ALD	390	Sn(acac)_2_ + H_2_S	–	818	10^4^	–	[226]
	Two‐step ALD	90/240	Sn(dmamp)_2_ + H_2_S	–	0.18	80	–	[236]
SnSe	ALD	390	Sn(acac)_2_ + H_2_Se	–	10	≈10^5^	–	[237]
PbSe	PS‐cALD	RT	PbCl_2_ + Na_2_Se	4.7	7.5 × 10^−3^	10^3^	–	[238]

^a)^ Note that HMDST is hexamethyldisilathiane; DEDS is diethyl disulfide; WH_2_(^i^PrCp)_2_ is bis(isopropyl cyclopentadienyl) tungsten(IV) dihydride; Sn(amd)_2_ is bis(N, N′‐diisopropylacetamidinato) tin(II); Sn(acac)_2_ is tin(II) acetylacetonate

^b)^ This value means Hall mobility.

### 3.1 Molybdenum Dichalcogenides

Molybdenum disulfide (MoS_2_), which occurs naturally as a mineral (molybdenite), has attracted great attention in high‐performance FETs due to its tunable intrinsic bandgap (1.2–1.9 eV) and high carrier mobility (up to 500 cm^2^ V^−1^ s^−1^).^[^ ^209^, ^239^ ^]^ The first publication of single‐layer MoS_2_ used within transistors reported excellent semiconductor properties with a mobility of 200 cm^2^ V^−1^ s^−1^ and an *I*_ON_/*I*_OFF_ of 10^8^ at room temperature.^[^ ^240^ ^]^ Subsequent demonstrations of mono‐to‐few layer MoS_2_ transistors further supported the initial findings.^[^ ^241^, ^242^, ^243^ ^]^

Numerous MoS_2_ precursor combinations have been used in the ALD of TMCs such as MoCl_5_ and H_2_S,^[^ ^218^, ^244^ ^]^ Mo(CO)_6_ and H_2_S,^[^ ^245^ ^]^ Mo(CO)_6_ and CH_3_S_2_CH_3_,^[^ ^246^ ^]^ Mo(NMe_2_)_4_ and H_2_S,^[^ ^73^ ^]^ as well as Mo(NMe_2_)_2_(N^t^Bu)_2_ and H_2_S.^[^ ^63^ ^]^ Benefiting from the layer‐controllability and reproducibility, ALD‐MoS_2_ shows great potential for applications in FETs.^[^ ^209^, ^219^ ^]^ For example, MoS_2_ channel fabricated by ALD from MoCl_5_ displays an effective mobility of 1.0 cm^2^ V^−1^ s^−1^ in back‐gate FETs with a 20 µm channel.^[^ ^221^ ^]^ However, temperature requirements of the MoCl_5_‐based process are beyond the upper limit tolerated by organic polymer substrates and photoresists.^[^ ^73^ ^]^ Additionally, the generation of chlorine‐containing by‐products such as HCl results in etching damage to both substrates and deposited films.

**Figure 16:**
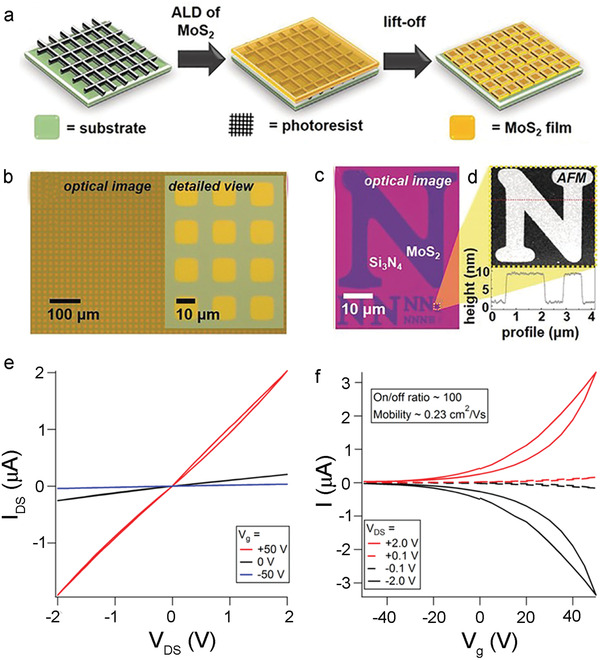
a) Schematic of a low‐temperature ALD‐MoS_2_ process with lithographic lift‐off patterning. b,c) Optical images of low‐temperature deposited MoS_2_ squares (b) and MoS_2_ letters (c) on Si/Si_3_N_4_ patterned by electron‐beam lithography. d) AFM image and height profile of MoS_2_ letters. e) Output and f) transfer curves of the annealed ALD‐MoS_2_ film. Reproduced with permission.^[^ ^73^ ^]^ Copyright 2017, Wiley‐VCH.

Metal amides show promise as metal precursors for low‐temperature ALD due to their high reactivity and generation of volatile byproducts.^[^ ^63^, ^73^ ^]^ By adopting Mo(NMe_2_)_4_ as a metal precursor and H_2_S as a co‐reactant, ALD‐MoS_2_ films were deposited at a record low reaction temperature of 60 °C.^[^ ^73^ ^]^ Low ALD temperatures enable simple device fabrication using lithographic lift‐off patterning (**Figure** **16**a). First, a silicon nitride‐coated silicon wafer is covered by a patterned resist using a mask‐less lithography protocol. The MoS_2_ film of the desired thickness is then deposited by ALD at 80 °C. Removal of the resist using n‐methyl‐2‐pyrrolidone (NMP) at 75 °C yields uniform arrays of patterned MoS_2_ (Figure 16b–d). Finally, post‐deposition sulfurization is performed under an atmosphere of sulfur vapor for 5 h, yielding highly crystalline MoS_2_. The electrical characteristics of the MoS_2_ semiconductor are further evaluated as FET components (Figure 16e,f), delivering a mobility of 0.23 cm^2^ V^−1^ s^−1^ and an *I*_ON_/*I*_OFF_ of ≈10^2^.

However, the low temperature used to deposit MoS_2_ results in amorphous films with numerous defects, leading to poor FET performance observed in the large off‐state current and low mobility.^[^ ^239^ ^]^ Improved crystallinity and better electrical performance of TMC films may be achieved using post‐deposition sulfurization of the film at high temperatures.^[^ ^239^, ^247^ ^]^ The standard sulfur sources such as sulfur powder and H_2_S,^[^ ^219^, ^248^ ^]^ may also be replaced by carbon disulfide (CS_2_), a strong sulfurizing reagent with low toxicity and cost.^[^ ^239^, ^249^ ^]^ Moreover, the relatively low sulfurization temperature used for CS_2_ may be more compatible with electronic devices, especially for flexible transistors. Compared with as‐deposited ALD‐MoS_2_, improved electrical performance was demonstrated by sulfurization using CS_2_ to obtain ≈100 times higher *I*_ON_/*I*_OFF_ (5 × 10^2^) and 36 times higher mobility (0.36 cm^2^ V^−1^ s^−1^).^[^ ^239^ ^]^

**Figure 17:**
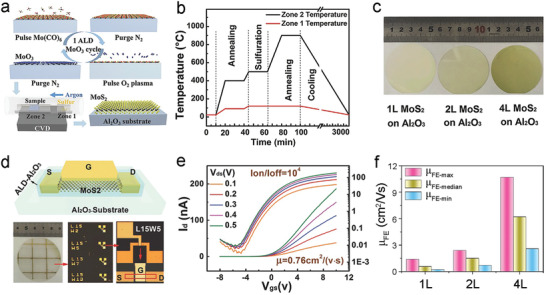
a) Schematic of the process for preparing thin‐film MoS_2_ by sulfurizing ALD‐MoO_3_. b) Temperature profiles of the CVD sulfurization process. c) Photo of large area single‐, bi‐ and tetra‐layer MoS_2_ films on a 2‐inch sapphire. d) Schematic device and optical images of FETs based on single‐layer MoS_2_. e) Transfer curves of the FETs based on the single MoS_2_ film. Reproduced with permission.^[^ ^219^ ^]^ Copyright 2017, Wiley‐VCH. f) Comparison in field‐effect mobility of FETs with different layer numbers of the ALD‐derived MoS_2_ channel, where these data were collected from the Ref. [219].

Sulfurizing MoO_3_ films pre‐deposited by ALD is another possible technique to synthesize high‐quality MoS_2_ films with controlled thicknesses.^[^ ^219^, ^250^, ^251^ ^]^ Sulfurization of the ALD‐MoO_3_ film at 500 °C followed by annealing at 900 °C with sulfur powder yields a MoS_2_ film (**Figure** **17**a,b).^[^ ^219^ ^]^ The layer thickness of MoS_2_ may also be precisely adjusted from monolayer to four layers (Figure 17c). When used to prepare a top‐gate FET (Figure 17d), the monolayer MoS_2_ film shows a typical n‐type FET behavior with a mobility of 0.76 cm^2^ V^−1^ s^−1^ and an *I*_ON_/*I*_OFF_ of 10^4^ (Figure 17e). By contrast, the electron mobility of the tetra‐layer‐MoS_2_ (5.9 cm^2^ V^−1^ s^−1^) is more than 8 times higher than that of the monolayer MoS_2_ (Figure 17f). Similar trends are observed for CVD‐MoS_2_ with mobility values increasing from 3.6 to 15.6 cm^2^ V^−1^ s^−1^ in the monolayer and trilayer, respectively.^[^ ^252^ ^]^ The improved performance of MoS_2_ may be due to the enhanced screening effect induced by an increase in layer numbers of MoS_2_.^[^ ^253^ ^]^ Generally, monolayer MoS_2_ suffers from fluctuating potential, which is caused by the formation of traps at the interface between the dielectric layer and the semiconductor channel.^[^ ^252^ ^]^ The additional layers of TMCs can efficiently suppress the negative effects of potential fluctuation, thus resulting in a higher carrier mobility.

Although previous reports have presented deposition of TMCs on various substrate materials, it remains challenging to synthesize wafer‐scale, uniform 2D TMCs in a scalable manner via direct ALD routes.^[^ ^209^, ^219^ ^]^ This obstacle hinders the integration of 2D TMCs for practical applications in the semiconductor industry. Thus, advances in ALD protocols to produce large‐scale, homogeneous 2D TMC thin films are necessary. Consequently, Jeon et al. have synthesized homogeneous few layers of MoS_2_ on a 6‐inch wafer by ALD with the utilization of diethylsulfide (DES) as an inhibitor layer.^[^ ^209^ ^]^ The deposited MoS_2_ films exhibited a significant increase in grain size and surface coverage (>620%), as well as excellent room temperature mobility (13.9 cm^2^ V^−1^ s^−1^) and *I*_ON_/*I*_OFF_ (>10^8^).

**Figure 18:**
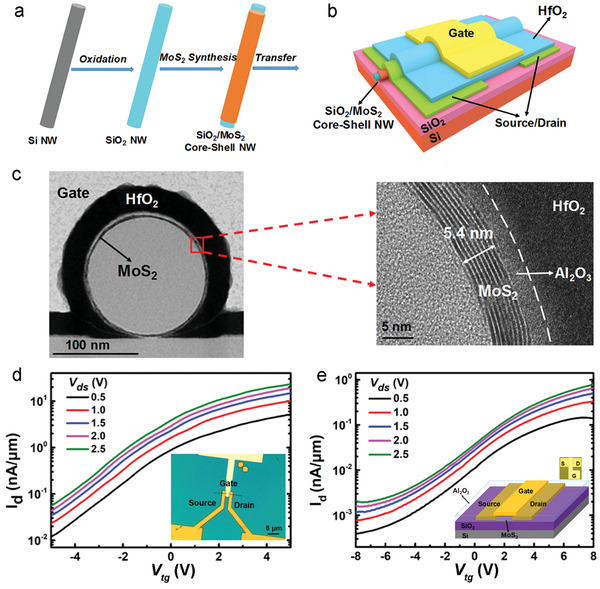
a) ALD‐assisted fabrication process of an omega‐shaped SiO_2_/MoS_2_ core‐shell nanowire heterostructure. b) Schematic and c) cross‐section TEM image of a FET based on the SiO_2_/ALD‐MoS_2_ heterostructure. d, e) Transfer curves of FETs based on the SiO_2_/MoS_2_ core‐shell nanowire heterostructure (d) and on a planar MoS_2_ channel (e). The insets are the corresponding FET structures for device testing. Reproduced with permission.^[^ ^224^ ^]^ Copyright 2020, American Chemistry Society.

Conformal surface coating of TMC films onto nonplanar, large surface‐to‐volume ratio substrates has been extensively demonstrated using ALD, highlighting the benefits to flexible electronic applications.^[^ ^224^, ^227^, ^233^ ^]^ Deposition of an ultrathin MoS_2_ layer onto a SiO_2_ nanowire via ALD (**Figure** **18**a) affords a core‐shell nanowire FET architecture (Figure 18b) with an omega (Ω)‐shaped top gate.^[^ ^224^ ^]^ Cross‐sectional TEM images of the deposited nonplanar MoS_2_ (Figure 18c) highlight the formation of uniform and high‐crystalline MoS_2_ layers. The fabricated Ω‐shaped MoS_2_ FETs exhibit a mobility of 0.02 cm^2^ V^−1^ s^−1^ and an *I*_ON_/*I*_OFF_ of ≈10^2^ (Figure 18d), which are comparable to those of the planar MoS_2_ FETs with a mobility of 0.01 cm^2^ V^−1^ s^−1^ and an *I*_ON_/*I*_OFF_ of ≈3.5 × 10^2^ (Figure 18e).

In comparison with MoS_2_, molybdenum selenide (MoSe_2_) displays a similar high average room temperature mobility (≈50 cm^2^ V^−1^ s^−1^) and a high *I*_ON_/*I*_OFF_ ratio (10^6^) in addition to bandgaps ranging from 1.1 to 1.6 eV.^[^ ^254^ ^]^ MoSe_2_ materials are typically prepared by exfoliation and CVD.^[^ ^254^, ^255^ ^]^ However, ALD techniques may also deposit MoSe_2_ using Mo(CO)_6_ or MoCl_5_ and ((CH_3_)_3_Si)_2_Se as molybdenum and selenium precursors, respectively.^[^ ^256^ ^]^ Selenization of ALD‐MoO_3_ also achieves high‐quality MoSe_2_ films with the desired thickness.^[^ ^257^, ^258^ ^]^ Similarities between silicon and MoSe_2_, including bandgap size and electronic properties, make the latter an excellent candidate for application as a semiconductor channel material.

Molybdenum ditelluride (MoTe_2_) possesses a smaller bandgap (1.1 eV for monolayer and 0.88 eV for multi‐layers) than its lighter chalcogenide analogs, but exhibits unique ambipolar‐type FET properties.^[^ ^259^, ^260^, ^261^ ^]^ Fabrication of MoTe_2_ films may be accomplished through direct ALD methods,^[^ ^262^ ^]^ or post‐deposition tellurization of ALD‐MoO_3_ films.

### 3.2 Tungsten Dichalcogenides

Tungsten disulfide (WS_2_), another representative of TMCs, exhibits an indirect bandgap of 1.4 eV (bulk) and a direct bandgap of 2.1 eV (monolayer).^[^ ^263^ ^]^ It shows great potential in FET applications due to its high theoretical mobility.^[^ ^264^ ^]^ WS_2_‐based FETs have been predicted with the highest mobility of over 1100 cm^2^ V^−1^ s^−1^ in a monolayer at room temperature.^[^ ^265^ ^]^ Up to now, WS_2_ has been successfully prepared by ALD with WF_6_ and H_2_S,^[^ ^266^, ^267^, ^268^ ^]^ tungsten chlorides (WCl_5_ and WCl_6_) and H_2_S,^[^ ^226^, ^269^ ^]^ W(CO)_6_ and H_2_S,^[^ ^270^ ^]^ W(NMe_2_)_2_(N^t^Bu)_2_ (BTBMW) and H_2_S,^[^ ^59^ ^]^ as well as sulfurizing thickness‐controlled ALD‐WO_3_ films.^[^ ^228^, ^229^ ^]^

**Figure 19:**
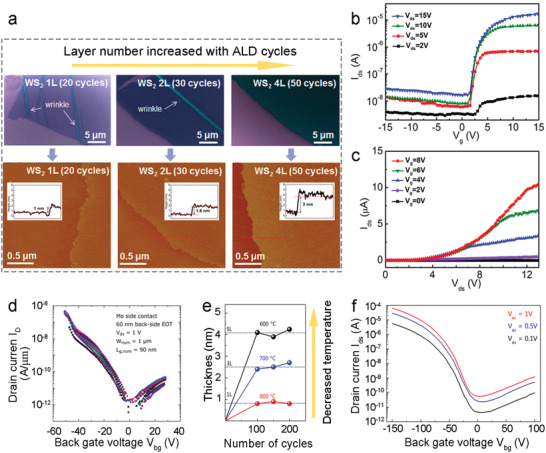
a) Optical (up) and AFM (down) images of the transferred single, bi‐ and tetra‐layer ALD‐WS_2_ nanosheet on SiO_2_ substrates. b) Transfer and c) output curves for the FET based on the single layer ALD‐WS_2_ film. Reproduced with permission.^[^ ^228^ ^]^ Copyright 2013, American Chemical Society. d) Transfer curves of FETs based on PEALD‐WS_2_ with p‐type characteristics. Reproduced with permission.^[^ ^227^ ^]^ Copyright 2018, American Chemical Society. e) Variance in film thickness of ALD‐WSe_2_ with controlled reaction temperatures of 600 (black), 700 (blue), and 800 °C (red) synthesized by a SLS method, respectively. f) Transfer curves of a FET based on three‐layer ALD‐WSe_2_. Reproduced with permission.^[^ ^231^ ^]^ Copyright 2016, IOP Publishing Ltd.

Earlier reports have demonstrated that ALD‐WS_2_ can exhibit both n‐type and p‐type semiconducting characteristics.^[^ ^227^, ^228^ ^]^ For example, atomically thin WS_2_ films prepared by sulfurizing ALD‐WO_3_ operate as n‐type semiconductors with an electron mobility of 4.5 cm^2^ V^−1^ s^−1^ and an *I*_ON_/*I*_OFF_ of ≈10^5^.^[^ ^229^ ^]^ The thickness of ALD‐WO_3_ precisely controls the layer number of WS_2_ from monolayer to tetra‐layer (**Figure** **19**a).^[^ ^228^ ^]^ The WS_2_‐based top‐gate FET displays impressive transfer and output characteristics with a high electron mobility of 3.9 cm^2^ V^−1^ s^−1^ and a low SS of 0.6 V dec^−1^ (Figure 19b,c). In general, the impurity content as well as structural defects of deposited films significantly affects the electrical properties and even alter the semiconducting behaviors of 2D materials.^[^ ^209^ ^]^ For instance, PEALD‐WS_2_ with a large crystal grain size operates as a p‐type semiconductor with an *I*_ON_/*I*_OFF_ of ≈10^5^ (Figure 19d).^[^ ^227^ ^]^ It is speculated that p‐type behaviors of ALD‐WS_2_ FETs may arise from S deficiency in the WS_2_ films.

Currently, research into tungsten selenide (WSe_2_) for electronic applications is limited, although exfoliated single‐crystal WSe_2_‐based FETs have been demonstrated to exhibit a high hole mobility of 350 cm^2^ V^−1^ s^−1^ at 300 K and a high *I*_ON_/*I*_OFF_ of 10^6^.^[^ ^271^ ^]^ Park et al. prepared WSe_2_ by self‐limited layer synthesis (SLS) using WCl_6_ and diethyl selenide at a high temperature above 600 °C.^[^ ^231^ ^]^ Unlike conventional ALD processes, the layer number of target films prepared by this SLS process mainly depends on the reaction temperature rather than the SLS cycles. Control of the SLS temperature from 600 to 800 °C affords WSe_2_ films with a tunable layer number, from five‐layers to a monolayer (Figure 19e). Back‐gated FETs were used to evaluate a trilayer‐WSe_2_ film, exhibiting a hole mobility of 2.2 cm^2^ V^−1^ s^−1^ and an *I*_ON_/*I*_OFF_ of ≈10^6^ (Figure 19f).

The ambipolar characteristics of ALD‐WSe_2_ were further demonstrated on large area (5 × 5 cm^2^) silicon wafers in back‐gated FETs.^[^ ^230^ ^]^ High mobility for both n‐type (531 cm^2^ V^−1^ s^−1^) and p‐type (354 cm^2^ V^−1^ s^−1^) carriers were recorded with high *I*_ON_/*I*_OFF_ ratios (≈10^5^).

### 3.3 Tin Chalcogenides

Tin disulfide (SnS_2_) is an intrinsic n‐type semiconductor with a bandgap of 2.1 eV, larger than that of monolayer MoS_2_.^[^ ^272^ ^]^ The high bandgap suppresses source/drain tunneling in FETs, affording a high *I*_ON_/*I*_OFF_ of 10^8^.^[^ ^273^ ^]^ Furthermore, large electron mobility (230 cm^2^ V^−1^ s^−1^) was demonstrated using adsorbate‐suppressing conditions, indicating its potential for applications in FETs.^[^ ^274^ ^]^ The fabrication of SnS_2_ films by ALD has been demonstrated using TDMASn and H_2_S,^[^ ^122^, ^234^, ^275^ ^]^ bis(1‐dimethylamino‐2‐methyl‐2‐propoxy) tin(II) and H_2_S plasma,^[^ ^74^ ^]^ Sn(OAc)_4_ and H_2_S,^[^ ^276^ ^]^ as well as sulfurizing ALD‐SnO_x_.^[^ ^233^ ^]^ Phase engineering of precursor thin films via sulfurization methods has shown promising results. Back‐gated FET channels prepared via deposition of an ALD‐SnS film followed by a sulfurization step, performed as an n‐type semiconductor with small mobility (0.014 cm^2^ V^−1^ s^−1^).^[^ ^234^ ^]^ Such poor electrical properties of SnS_2_ can be improved by enhancing its crystallinity and control crystal orientation,^[^ ^233^ ^]^ as well as optimizing the gate structures.^[^ ^277^ ^]^

**Figure 20:**
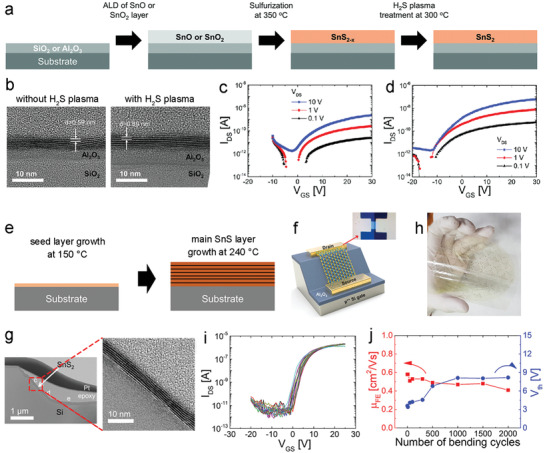
a) Illustration of the two‐step sulfurization for 2D SnS_2_. b) HRTEM images of the SnS_2_ on Al_2_O_3_ without (left) and with (right) further H_2_S plasma treatment. c,d) Transfer curves of the TFTs based on ALD‐derived SnS_2_ with the one‐step sulfurization (c) and the two‐step sulfurization (d), respectively. Reproduced with permission.^[^ ^233^ ^]^ Copyright 2018, Royal Society of Chemistry. e) A schematic two‐step ALD process for SnS_2_. f) Schematic of nonplanar SnS_2_ TFTs with a diagonal‐structure and g) cross‐sectional HRTEM images of the ALD‐derived SnS_2_ film. h) Optical image and i) transfer curves of the flexible SnS_2_ TFTs with 20 devices distributed across a PI substrate. j) Trend in mobility and *V*_T_ of flexible TFTs under different bending cycles. Reproduced with permission.^[^ ^74^ ^]^ Copyright 2020, American Chemical Society.

ALD‐SnO as a precursor for SnS_2_ was demonstrated using a two‐step sulfurization process, consisting of an initial thermal post‐deposition sulfurization (350 °C), and then an H_2_S‐plasma treatment (**Figure** **20**a).^[^ ^233^ ^]^ The SnS_2_ film with subsequent H_2_S plasma treatment shows better‐suited crystal orientation and improved crystallinity than that with simple sulfurization (Figure 20b). Back‐gate TFTs fabricated using these SnS_2_ films show typical n‐type behaviors (Figure 20c,d), where the *I*_ON_/*I*_OFF_ is greatly increased to 10^5^ for the TFTs with two‐step sulfurized SnS_2_, compared with 10^3^ for that with one‐step sulfurized SnS_2_. Similarly, the field‐effect mobility is also dramatically improved from 2 × 10^−4^ to 0.02 cm^2^ V^−1^ s^−1^.

A two‐step ALD process was demonstrated for obtaining crystalline and continuous SnS_2_ films at a low temperature.^[^ ^276^ ^]^ An amorphous SnS_2_ thin layer deposited at 150 °C was sulfurized at a relatively high temperature (250 °C), then the formed crystalline SnS_2_ acted as a seed layer for promoting the growth of crystalline SnS_2_ films by low‐temperature ALD (150 °C). Another two‐step ALD procedure demonstrated by Pyeon et al., applies low temperature (150 °C) deposition of monolayer amorphous SnS_2_ followed by a subsequent SnS_2_ thin channel layer (240 °C) (Figure 20e).^[^ ^74^ ^]^ The amorphous monolayer selectively promotes basal growth of SnS_2_ (001) during the second deposition, thus suppressing non‐uniform nuclei orientation. The relationship between surface energy and grain orientation was not explored but may offer an avenue for further research. Analysis of the electrical performance as a bottom‐gate TFT revealed that the as‐deposited SnS_2_ film mobility could be improved using an additional sulfurization step (300 °C), from 0.2 to 0.8 cm^2^ V^−1^ s^−1^. The excellent control of grain orientation made this procedure well suited for fabricating non‐planar (Figure 20f) and flexible SnS_2_ TFTs (Figure 20h). Thus, direct deposition onto non‐planar substrates was undertaken to investigate the practical application of this method. Cross‐sectional TEM images (Figure 20g) of a diagonally constructed TFT highlight the excellent conformality and basal growth of the 2D SnS_2_ channel layer. The mobility and *I*_ON_/*I*_OFF_ display moderate outputs of 0.4 cm^2^ V^−1^ s^−1^ and ≈10^6^ for the nonplanar ALD‐SnS_2_ TFTs after post‐annealing, respectively. The flexible ALD‐SnS_2_ TFTs exhibit stable electric performance with a mobility of 0.57 ± 0.02 cm^2^ V^−1^ s^−1^ and an *I*_ON_/*I*_OFF_ of 10^6^ (Figure 20i). After 2000 bending cycles at a radius of 17.5 mm, the flexible TFTs show slight performance degradation (Figure 20j). These results highlight the practicality of ALD for the preparation of flexible and nonplanar MC devices.

In contrast to SnS_2_, tin monosulfide (SnS) is intrinsically p‐type, with a small indirect bandgap (1.0 eV).^[^ ^122^, ^235^ ^]^ ALD synthesis affords p‐type SnS films with a hole mobility of 0.21 cm^2^ V^−1^ s^−1^ but a relatively small *I*_ON_/*I*_OFF_ of 8.8.^[^ ^234^ ^]^ Generally, SnS films with high‐crystal orientation can be synthesized at a relatively low ALD temperature,^[^ ^235^ ^]^ which is beneficial for the fabrication of electronic devices.^[^ ^73^, ^122^ ^]^ For instance, the Hall mobility of SnS‐based FETs was improved from 0.82 to 15.3 cm^2^ V^−1^ s^−1^ by tuning ALD reaction temperatures.^[^ ^235^ ^]^ The significant improvement in carrier transport is ultimately attributed to the preferred crystal orientation of the ALD‐SnS film.

The purity of channel materials is also an essential parameter that cannot be ignored in evaluating the influence on electrical performance.^[^ ^278^ ^]^ In some cases, multiple phases of SnS_x_ may be present, including mixtures of SnS, SnS_2_, and Sn_2_S_3_, which may seriously limit electrical properties.^[^ ^122^, ^278^ ^]^ However, conversion of the as‐deposited SnS_x_ mixtures to a single‐phase SnS film may be achieved via post‐deposition annealing under H_2_ (360 °C), contributing to a greatly improved Hall mobility of 15.66 cm^2^ V^−1^ s^−1^ compared with the as‐deposited SnS_x_ film of 2.83 cm^2^ V^−1^ s^−1^.^[^ ^278^ ^]^

**Figure 21:**
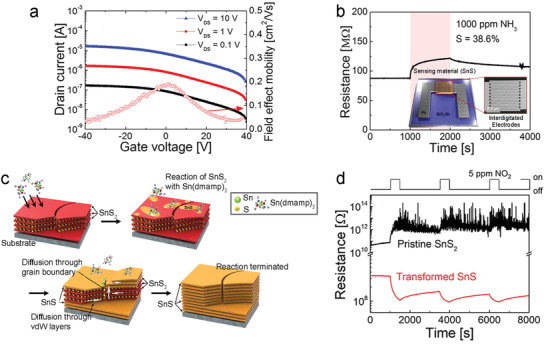
a) Transfer curves of the ALD‐SnS TFT. b) Gas response of the ALD‐SnS based sensing device under exposure to 1000 ppm NH_3_ at room temperature (the inset is a schematic gas sensor with an SEM image of its interdigitated electrodes). Reproduced with permission.^[^ ^236^ ^]^ Copyright 2017, American Chemical Society. c) Illustration of the transformation process from ALD‐SnS_2_ to ALD‐SnS with the assistance of Sn(dmamp)_2_. d) Gas response of the bottom‐gate TFT gas sensor based on pristine ALD‐SnS_2_ (black) and transformed ALD‐SnS (red) under exposure to 5 ppm NO_2_ at room temperature. Reproduced with permission.^[^ ^279^ ^]^ Copyright 2020, American Chemical Society.

An analogous two‐step strategy has also been applied for the ALD of SnS semiconductors.^[^ ^236^ ^]^ A continuous amorphous ALD‐SnS film was previously deposited at lower temperatures (90 °C), followed by a high‐temperature deposition of a crystalline SnS at 240 °C. The SnS TFTs exhibit p‐type behaviors with a mobility of 0.18 cm^2^ V^−1^ s^−1^ and an *I*_ON_/*I*_OFF_ ratio of 80 (**Figure** **21**a). Correspondingly, the ALD‐SnS gas sensors also display high sensitivity for NH_3_ detection (Figure 21b). The facile transformation of ALD‐SnS_2_ to SnS with the assistance of Sn(dmamp)_2_ vapors is also feasible (Figure 21c).^[^ ^279^ ^]^ Sn(dmamp)_2_ molecules adsorb onto the SnS_2_ surface, triggering a reduction of the Sn(IV) forming SnS and volatile byproducts. The opposing behaviors of bottom‐gate TFTs using SnS_2_ and SnS in response to NO_2_ gas sensing (Figure 21d) support the majority carrier species of the two phases are electrons and holes, respectively.

In addition to SnS_2_ and SnS, tin selenide (SnSe) shows promise as a low bandgap (0.9 eV for indirect and 1.3 eV for direct) layered nanomaterial for electronic applications.^[^ ^237^, ^280^ ^]^ Semiconducting ALD‐SnSe films can exhibit good p‐type behavior with a high hole mobility of 10 cm^2^ V^−1^ s^−1^ and an *I*_ON_/*I*_OFF_ of ≈10^5^.

### 3.4 Other Metal Chalcogenides

Aside from Mo, W, and Sn‐based MCs, the fabrication of alternative MC materials such as lead sulfide (PbS), manganese sulfide (MnS), and rhenium disulfide (ReS_2_) is possible using ALD,^[^ ^210^, ^281^, ^282^ ^]^ with their development for electronic applications attracting growing interest.^[^ ^238^, ^283^ ^]^ MnS, is a p‐type semiconductor with a wide bandgap of 3.7 eV, and it has been deposited with crystal phase‐control by ALD using bis(ethylcyclopentadienyl)Mn(II) (Mn(EtCp)_2_) and H_2_S precursors.^[^ ^281^ ^]^ The remarkable properties include an *I*_ON_/*I*_OFF_ of >10^6^ and a field‐effect hole mobility of 0.1 cm^2^ V^−1^ s^−1^, when applying *α*‐MnS in a top‐gate FET.^[^ ^283^ ^]^ Moreover, the MnS FETs without dielectric encapsulation show negligible degeneration of device performance after being exposed to air for 30 days, indicating their excellent air stability. Similarly, ReS_2_ films can be synthesized on large‐area substrates by ALD using ReCl_5_ and H_2_S precursors at a wide deposition temperature range between 120 and 500 °C.^[^ ^282^ ^]^ Their use as channel materials in FETs have displayed attractive electrical performances (i.e., an *I*_ON_/*I*_OFF_ of 10^6^ and a SS of 750 mV dec^−1^).^[^ ^284^ ^]^

**Figure 22:**
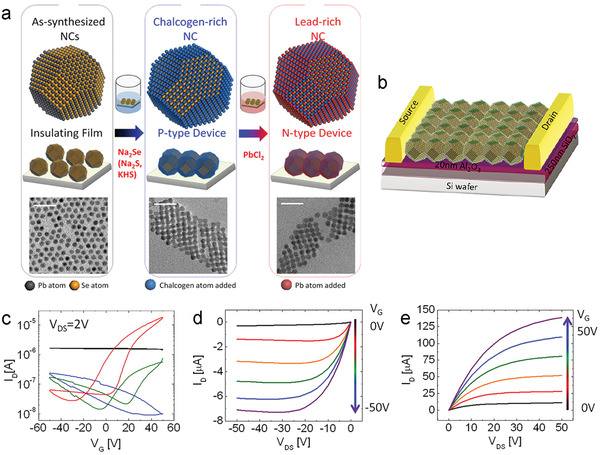
a) Schematic of PS‐cALD for preparing lead chalcogenide NC films and their corresponding TEM images. The left is an as‐synthesized NC thin‐film, the middle is a thin‐film treated with either Na_2_Se, Na_2_S or KHS solution, the right is a thin‐film with further treatment in PbCl_2_ solution. b) A schematic FET based on the channel of PbS or PbSe thin‐films. c) Transfer curves of Na_2_Se‐treated PbSe NCs without (black) and with 1 h (blue), 6 h (green), and 12 h (red) of PbCl_2_ treatment at 65 °C. d,e) Output curves of Na_2_Se‐treated PbSe with followed by further PbCl_2_ treatment for 1 h (d) and 12 h (e), respectively. Reproduced with permission.^[^ ^238^ ^]^ Copyright 2014, American Chemical Society.

Very recently, uniform and crystalline PbS thin‐films have been produced by low‐temperature ALD using rac‐N^2^,N^3^‐di‐tert‐butylbutane‐2,3‐diamide lead [Pb(dbda)] and bis(trimethylsilyl)‐amide lead [Pb(btsa)_2_] as lead precursors, with H_2_S as a sulfur source.^[^ ^210^ ^]^ The PbS films exhibited typical p‐type behaviors with excellent mobility up to 70 cm^2^ V^−1^ s^−1^. Lead chalcogenides are a well‐established class of ambipolar semiconductors, with carrier transport determined by an excess of either metal or chalcogen within the crystal structure. Thus, chalcogen‐ and lead‐rich materials possess p‐ and n‐type characteristics, respectively.^[^ ^238^ ^]^ Post‐synthesis colloidal ALD (PS‐cALD) applies spin‐casting (or other solution‐processing techniques) to generate nanocrystals (NCs) with precise size control, followed by NC surface modifications using an adapted ALD protocol. Selective stoichiometric enhancement of the NC thin film using ALD (**Figure** **22**a) was achieved through subsequent modification of the NC surface using chalcogen‐ or lead‐based salts dissolved into an organic solvent (65 °C).^[^ ^238^ ^]^ The electrical properties of the stoichiometry‐controlled lead chalcogenides were evaluated in a bottom‐gate FET (Figure 22b). The transfer curves of PbSe after post‐treatment with PbCl_2_ reveal the influence of increasing Pb‐surface stoichiometry on carrier transport as a function of time (Figure 22c). The initial PbSe NC FETs treated with Na_2_Se exhibit p‐type characteristics with a mobility of 7.5 × 10^−3^ cm^2^ V^−1^ s^−1^, which is decreased to 2.2 × 10^−3^ cm^2^ V^−1^ s^−1^ after 1h PbCl_2_ treatment. By extending the treatment time to 6 h, an ambipolar behavior appears in the FETs. After 12 h, n‐type NC FETs can be achieved with electron mobility as high as 4.5 cm^2^ V^−1^ s^−1^ and an *I*_ON_/*I*_OFF_ of ≈10^2^–10^3^. This transformation is also reflected in the output curves, as after 1 h PbCl_2_ post‐treatment, clear p‐type characteristics are observed (Figure 22d), whilst at 12 h n‐type characteristics are dominant (Figure 22e).

### 3.5 2D Heterostructures Based on MCs

In addition to single MC semiconductors, MCs‐based 2D heterostructures such as MoS_2_/graphene,^[^ ^222^ ^]^ MoS_2_/WSe_2_,^[^ ^225^ ^]^ and MoS_2_/WS_2_,^[^ ^285^ ^]^ may be constructed by stacking different 2D materials together.^[^ ^213^ ^]^ These heterostructures have been extensively studied in various applications including vertical tunneling FETs,^[^ ^286^ ^]^ photodetectors,^[^ ^222^ ^]^ and inverters.^[^ ^223^ ^]^ The low contact resistance within MC/graphene heterostructures affords improved performance in electronic devices,^[^ ^222^ ^]^ whilst TMC/TMC heterostructures can function as current rectifiers due to the formation of p‐n junctions.^[^ ^226^, ^287^ ^]^

**Figure 23:**
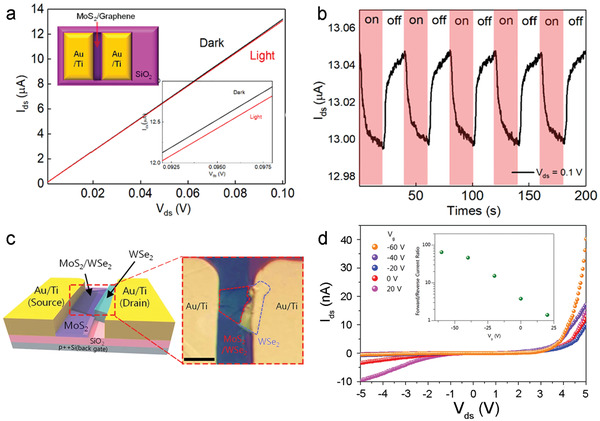
a) *I*_DS_‐*V*_DS_ curves and b) dynamic photoresponse of the ALD‐MoS_2_/graphene heterostructure‐based FETs. Reproduced with permission.^[^ ^222^ ^]^ Copyright 2019, Elsevier. c) Schematic device and optical image of the MoS_2_/WSe_2_ FET‐based PN diode, and d) the corresponding *I*‐*V* curves with various gate biases. Reproduced with permission.^[^ ^225^ ^]^ Copyright 2016, Nature Publishing group.

The deposition of single‐layer MoS_2_ directly onto graphene via ALD, combines properties of both materials in MoS_2_/graphene heterostructures to achieve a FET‐based photodetector.^[^ ^222^ ^]^ An *I*_DS_‐*V*_DS_ curve evaluates the fluctuation of potential in response to light (**Figure** **23**a), with a 116 nA photocurrent difference observed at a *V*_DS_ of 0.1 V and a responsivity of 241 mA W^−1^. The on/off switching behavior (Figure 23b) further demonstrates its optoelectronic applications, highlighting the potential of ALD‐derived 2D heterostructures.

Precursor selection is an important factor when fabricating 2D MC heterostructures using ALD, as by‐products such as HCl may result in surface etching. Thus, metal halide precursors using chloride ligands are typically avoided when used in conjunction with sensitive substrates, as is the case of MoS_2_/WSe_2_ heterostructures. The MoS_2_/WSe_2_ heterostructures have also been fabricated using the SLS process at 800 °C, where MoS_2_ was deposited onto exfoliated WSe_2_ flakes.^[^ ^225^ ^]^ As a result, a top‐gated FET device (Figure 23c) performs as a typical p‐n diode, showing rectifying characteristics with a forward/reverse current ratio of ≈80 at a *V*_G_ of −60 V (Figure 23d). Similarly, a p‐n diode based on InSe/Sb_2_Se_3_ heterostructure was also fabricated by stacking a p‐type Sb_2_Se_3_ layer on n‐type InSe, which were both deposited by ALD.^[^ ^287^ ^]^ The resulting heterostructure exhibited typical diode characteristics with a maximum leakage current of 10^−7^ A at −1 V bias.

The ALD of WS_2_/SnS heterostructures ensues to give an ambipolar thin film with electron mobility of 48 cm^2^ V^−1^ s^−1^ for n‐type FETs and a hole mobility of 20 cm^2^ V^−1^ s^−1^ for p‐type FETs at room temperature.^[^ ^226^ ^]^ However, the hole mobility of SnS in the heterostructure dropped significantly compared with that of the pure SnS (≈818 cm^2^ V^−1^ s^−1^). This result is attributed to hole transport resistance, which may be caused by the misalignment of the SnS and WS_2_ layers. The difference between crystal structures led to the growth of the SnS layer on WS_2_ at an orientation of ≈15°, resulting in a remarkable drop in the hole mobility of SnS. Thus, misalignment between different TMCs remains a continuing challenge for the fabrication of ALD heterostructures.

Although ALD is well‐suited to depositing MCs, there are still limited studies about the electrical applications of ALD‐MCs. Moreover, reports on constructing 2D MC heterostructures by ALD in electronics are also limited.^[^ ^222^, ^226^, ^287^ ^]^ Considering the attractive features of nanoscale MCs, 2D ALD‐MCs and their related heterostructures will play an important role in the development of next‐generation electronic devices. Additionally, multinary MCs with tunable properties and improved performance are also worthy of in‐depth studies. For example, Mo_1‐x_W_x_S_2_ exhibits a tunable optical bandgap from 1.87 to 2.00 eV, and displays an improved photocurrent relative to MoS_2_ and WS_2_, respectively.^[^ ^288^ ^]^ Continued research should focus on the broad applications and possibilities provided by ALD‐MCs and their derived architectures for advanced electronics and optoelectronics.

## 4 Conclusions and Perspective

In summary, this review has outlined recent advances in the fabrication of FET materials using ALD, as well as assessed their corresponding performances as integrated devices. The well‐established merits of ALD yielded FET materials with atomically precise layer thickness, whilst maintaining conformality to numerous substrate/device architectures. The large variety of key FET materials fabricated using ALD includes semiconductor channels, dielectrics, passivation/encapsulation layers, electrodes, and electrode interlayers, highlighting the benefits of ALD for FET manufacturing. The continuously expanding library of ALD precursors has enabled numerous MOs and MCs to be deposited as ultrathin films, whilst additional ALD factors facilitate control over stoichiometry, structure and bandgap properties. These factors, in conjunction with electronic performance metrics such as the charge carrier mobility, on/off current ratio, switching speed and device stability, enable the rapid and successive optimization of new materials for applications in FETs and their derived devices. Several wide/medium bandgap n‐ and p‐type MO have been fabricated as channel materials as well as transparent electrodes and electrode interlayers, resulting in improved charge transport and signal switching. Large bandgap MOs have also served as effective gate dielectrics or protecting/encapsulating layers for enhancing device performance, particularly the durability and operational stability. The rapid development of 2D materials and devices has also triggered investigation on the design of ALD‐MCs as promising channel materials for n‐type and p‐type transistors. These narrow/medium‐bandgap semiconductors are used as single channel layers as well as heterostructures for promoting charge transport, photodetection, gas sensing, and more.

Despite significant progress, ALD application still requires the development of cost‐effective precursors and methods, to further promote the integration of FET materials in high‐performance devices. Previous reports have focused on n‐type MO and MC semiconductors due to the absence of p‐type and bipolar counterparts. Impressively, reports of some p‐type semiconductors derived from ALD, including SnO, CuO, Cu_2_O, WS_2_ and WSe_2_, SnS and SnSe, as well as InSe, highlight the growing progress of p‐type ALD materials. Examples such as ZnO, TiO_2_, and lead chalcogenides may also display a transition to p‐type characteristics through appropriate doping with heteroatoms, substrate selection, and enrichment with metal elements. However, further research on p‐type materials using ALD is required for a more detailed evaluation of their electric, optoelectronic and mechanical properties, as well as their device stability (e.g., operational stability, air stability, photostability, bending stability, and so forth).

The capacity for ALD to tune the composition of MOs and MCs makes it well adapted to explore multinary FET materials. By controlling ALD reaction parameters (e.g., temperature, time, precursor, substrate, and pre‐/post‐treatment, etc.), thin‐film fabrication according to a desired stoichiometric composition affords facile optimization possibilities for the bandgap, mobility, and other physical/chemical properties. Moreover, ALD has the potential to fabricate more complex heterostructures in a manner that is not accessible with other techniques, but currently remains underexplored. For example, simple alternating bilayers of MO insulators have proven an effective adaptation for shielding devices against atmospheric water and oxygen surface adsorption. Design of such heterostructure using ALD is a promising strategy for boosting the electrical performance of FET devices using MO/MO, MC/MC, and hybrid MO/MC architectures. In addition, the uniqueness of ALD may also provoke greater integration of ALD techniques with other established methods (i.e., 3D printing, inject printing, flexographic printing, solution‐process patterning, and laser processing, etc.) towards advanced FET design and fabrication, which may prove useful for developing future micro‐/nano‐scale structures and devices.

The successful demonstration of ALD of MOs and MCs in high‐performance FETs reflects their promising potential as future components within electronic devices such as inverters, oscillators, and integrated circuits. In addition, other ALD‐derived materials, such as III‐V group semiconductors, metal nitrides, metal halides, perovskites, and hybrids, also show significant potential in advanced electronics and semiconductor‐integrated systems. However, current researches on most ALD‐materials still mainly focus on individual FET fabrication and is limited to laboratory‐scale. Meanwhile, translation of the ALD processes to device arrays based on FETs is an important step for developing the technology for larger‐scale manufacturing. This demand may stimulate the adoption of ALD‐FETs within newly emerging technologies such as multifunctional sensing, artificial synapses, self‐driving systems, smart manufacturing as well as an intelligent medical diagnosis and health monitoring. The emerging ALD processes of MOs and MCs with tunable compositions, structures, and electronic properties can provide the possibility to optimize optoelectronic and mechanical properties and promote widespread applications within electronics, photonics, information, and Internet of Things. Nowadays, the ever‐developing uses of artificial intelligence (AI) are accelerating the discovery and deployment of advanced materials as well as functional devices.^[^ ^289^ ^]^ Thus AI techniques such as machine learning and deep learning may be used to further develop ALD‐derived materials and FETs/chips for flexible circuits, actuators, intelligent robotics, integrated networks of quantum devices, wearable applications, and human‐machine interactions, etc. Moreover, the electronic information industry continues to gradually favor low‐power electronic devices as a strategy to reduce carbon emissions and achieve global carbon neutrality.^[^ ^290^ ^]^ Therefore, emphasis must be placed on the fabrication of electronic materials and devices by ALD, that match the growing global demands for green and sustainable electronics. The coming decades will continue to witness the rapid development in both improved ALD technologies and the availability of diverse MOs, MCs, and other emerging materials for new‐generation devices and realistic applications.

## Conflict of Interest

The authors declare no conflict of interest.
